# A comprehensive evaluation of binning methods to recover human gut microbial species from a non-redundant reference gene catalog

**DOI:** 10.1093/nargab/lqab009

**Published:** 2021-03-01

**Authors:** Marianne Borderes, Cyrielle Gasc, Emmanuel Prestat, Mariana Galvão Ferrarini, Susana Vinga, Lilia Boucinha, Marie-France Sagot

**Affiliations:** MaaT Pharma, 317 Avenue Jean Jaurès, 69007 Lyon, France; Université de Lyon, Université Lyon 1, CNRS, Laboratoire de Biométrie et Biologie Évolutive UMR 5558, F-69622 Villeurbanne, France; Erable team, INRIA Grenoble Rhône-Alpes, 655 Avenue de l’Europe 38330 Montbonnot-Saint–Martin, France; MaaT Pharma, 317 Avenue Jean Jaurès, 69007 Lyon, France; MaaT Pharma, 317 Avenue Jean Jaurès, 69007 Lyon, France; Université de Lyon, Université Lyon 1, CNRS, Laboratoire de Biométrie et Biologie Évolutive UMR 5558, F-69622 Villeurbanne, France; INSA-Lyon, INRA, BF2i, UMR0203, F-69621 Villeurbanne, France; INESC-ID, Instituto Superior Técnico, Universidade de Lisboa, 1000-029 Lisbon, Portugal; MaaT Pharma, 317 Avenue Jean Jaurès, 69007 Lyon, France; EVOTEC ID (Lyon), 40 Avenue Tony Garnier, 69007 Lyon, France; Université de Lyon, Université Lyon 1, CNRS, Laboratoire de Biométrie et Biologie Évolutive UMR 5558, F-69622 Villeurbanne, France; Erable team, INRIA Grenoble Rhône-Alpes, 655 Avenue de l’Europe 38330 Montbonnot-Saint–Martin, France

## Abstract

The human gut microbiota performs functions that are essential for the maintenance of the host physiology. However, characterizing the functioning of microbial communities in relation to the host remains challenging in reference-based metagenomic analyses. Indeed, as taxonomic and functional analyses are performed independently, the link between genes and species remains unclear. Although a first set of species-level bins was built by clustering co-abundant genes, no reference bin set is established on the most used gut microbiota catalog, the Integrated Gene Catalog (IGC). With the aim to identify the best suitable method to group the IGC genes, we benchmarked nine taxonomy-independent binners implementing abundance-based, hybrid and integrative approaches. To this purpose, we designed a simulated non-redundant gene catalog (SGC) and computed adapted assessment metrics. Overall, the best trade-off between the main metrics is reached by an integrative binner. For each approach, we then compared the results of the best-performing binner with our expected community structures and applied the method to the IGC. The three approaches are distinguished by specific advantages, and by inherent or scalability limitations. Hybrid and integrative binners show promising and potentially complementary results but require improvements to be used on the IGC to recover human gut microbial species.

## INTRODUCTION

The human gut microbiota represents one of the densest microbial environments harboring approximately 10^13^ microbial cells (1) and housing a few hundred microbial species in a healthy individual (2), including bacteria, phages, archaea and microeukaryotes. It exerts several functions essential to the maintenance of host physiology, such as prevention from colonization by pathogens (3), stimulation of the immune system (4) and metabolic regulation (5). The gut microbiota and its host maintain a complex symbiotic relationship known to be strongly associated with host health states and a disruption in the composition of the microbiota is observed in many diseases (6). Understanding how these different microorganisms play a role in human health is thus of crucial interest. However, characterizing the functioning of microbial communities in relation to the host remains a challenge. This is notably due to the small percentage of cultivable microorganisms in the gut microbiota, making the use of quantitative metagenomics indispensable to further explore the composition and diversity of microbial communities by giving access to the genes and genomes of uncultivated species (7).

The human gut microbiome has been well-studied within the context of whole-genome and whole-metagenome shotgun (WMS) sequencing projects whose purpose is to build reference microbial genome sequences and gene catalogs (2,8–10). Major international projects such as MetaHIT (2) and HMP (10) have thus permitted to build such gene catalogs to facilitate the analysis and understanding of the gut microbiota. However, in gene catalogs, a link between microbial genes and species is not yet established. Indeed, taxonomic (genome-based) and functional (gene-based) analyses are performed independently because 40–50% of the human gut microbial species are not represented by genomes from cultured isolates (11,12). To overcome this issue, recent studies have established a collection of genomes based on large-scale cultivation, or from the recovered individual genomes of thousands of gut metagenomes (13–18). Their recovery was achieved by performing *de novo* assembly of WMS reads and by binning the assembled contigs into Metagenome-Assembled Genomes (MAGs) for each sample. As for functional analyses, reference gene catalogs remain an essential and well-established resource (2). Although the reconstruction of MAGs has become very popular in the past years, and has even recently resulted in a protein catalog (18), contig-binning performed sample-by-sample has also demonstrated drawbacks that can be addressed by gene-binning of large non-redundant catalogs, including (i) the exclusion of short assembled sequences, below 1000 bp at most (15–17) which can correspond to genes or partial genes; (ii) biases preventing the reconstruction of low-abundance organisms (15), notably due to insufficient genome coverage. Furthermore, gene-binning allows to identify and accurately quantify the abundance of species-specific marker genes; hence, it provides a more straightforward approach than MAGs for the estimation of species relative abundance. With the aim to characterize complex microbial communities and to provide a structured reference for metagenome-wide association studies (19), Nielsen *et al.* (20) proposed Canopy (mgs-canopy), a method to bin co-abundant genes across metagenomic samples into species-level bins, called metagenomic species (MGS), and applied it to an extended version of the MetaHIT gene catalog. MGS allow to perform functional and taxonomic analyses by mapping reads from metagenomic samples onto the catalog of genes, thereby associating sample reads to metabolic functions and to one or several bins representing known or unknown species. The Integrated Gene Catalog (8) (IGC) later became the most used publicly available catalog for such analyses. It is composed of 9.9 million non-redundant genes and was built from 1267 human gut microbiome samples. However, even though a recently published binning method (binner) named mspminer (21) has been applied to the IGC, there is no consensus on an established reference species-level bin set for this reference catalog.

Several recently published and reviewed taxonomy-independent binners (also called reference-free binners) have been proposed, implementing different approaches either based on the co-abundance of sequences (abundance-based), on their abundance and composition (hybrid) or on the integration of the results from multiple binning methods (integrative). Binning reviews claim that hybrid binners (22,23) and integrative methods (24) are more likely to achieve better results. Published benchmarking results such as the first Critical Assessment of Metagenome Interpretation (CAMI) challenge (25,26) have highlighted best-performing binners, some of which have been afterwards used for the reconstruction of MAGs (15–17). However, they do not include methods based on binning co-abundant genes and the focus of these benchmarking results is on the reconstruction of individual genomes from the assembled contigs of a metagenome. Moreover, apart from Plaza Oñate *et al.* (21), mgs-canopy and mspminer (both abundance-based) have not been benchmarked together. To properly recover species-level bins from a reference non-redundant gene catalog, there are at least three essential particularities to take into account (i) the lengths of the sequences to cluster which tend to be short; (ii) the fact that a microbial gene can be shared by several species; and (iii) the increasing and relatively large number of genes and of samples to process.

In this study, we aim to identify the most suitable approach to cluster a large non-redundant set of genes into species-level bins, and more specifically the genes of the IGC. To this purpose, we designed a simulated non-redundant gene catalog (SGC) composed of genes from 41 microbial species, most of which are associated with the human gut microbiota. We then selected published and reviewed taxonomy-independent binners implementing distinctive approaches which are complementary to the abundance-based approaches that have already been applied to the IGC. Therefore, the benchmarking on our SGC comprises nine binners including two abundance-based, six hybrid and one integrative method. The selected binners of these last two categories were designed to recover genomes from contigs, hence they are used in our study in a different context than the one they were initially developed for and hybrid binners expect relatively long sequences (generally at least 1000 bp). Considering the particularities of the SGC and the IGC, we adapted the quality assessment tool amber (26) to evaluate binners on a non-redundant set of genes (i.e. where a gene can be assigned to one or to multiple bins). In addition to the adjustment of standard binning quality assessment metrics, we computed metrics specific to covers (i.e. overlapping clusters). In order to further explore the three binning approaches, we compared the results of the best-performing binner of each category with our expected community structures (species and strain levels), taking into account the characteristics of the genes (e.g. 16S ribosomal RNA–16S rRNA, prophage and plasmid genes) and corresponding genomes (e.g. GC content, closely related genomes) included in our SGC. Finally, we evaluated the usability and scalability of the best-performing binners on the IGC. We believe that our results as well as our methodology will be of great use to the community to gain insight into which binner is the most adequate to handle specific cases, thereby to bin the sequences of a given dataset such as a non-redundant microbial gene catalog, and on how to evaluate overlapping binning results.

## MATERIALS AND METHODS

### Selected taxonomy-independent binners

We benchmarked nine binners classified into three categories of approaches (Table 1): abundance-based, hybrid and integrative. Considering the large number of available binners, in our selection, we have only included binners that have been published and reviewed at the time of this study. Given that our aim is to evaluate the suitability and usability of the main current approaches to cluster a large non-redundant set of genes into species-level bins, we selected binners implementing distinctive and complementary approaches to the ones already applied to the IGC. We included two abundance-based (mgs-canopy (20) and mspminer (21)), six hybrid (solidbin (27), cocacola (28), concoct (29), metabat (30), maxbin (31) and mycc (32)), and one integrative (das tool (33)) binner. More information on our selection criteria is provided in the Supplementary Data.

**Table 1.** Selected taxonomy-independent binners. Binners are organized into three categories based on their approach: abundance-based (Ab.), hybrid (Hyb.), integrative (Int.). For abundance data, we used the raw or reformatted (e.g. sorted, split or filtered) intermediary or final files generated by mocat as input for each binner. Among the different profiles generated by mocat, we used the one we assessed the most appropriate based on the publication or user guide of each binner. Non-default information on the selected runs are specified such as the mode, parameters settings, filters applied to the input files, or the selected set of final bins. More information on the number of used threads is available in the Supplementary Data. We ran each hybrid binner with the default value for the gene length filter and lowered it down to 500 bp when proposed by the tool, and when the computational requirements were reasonable. It should be noted that metabat can include short genes (<1500 bp and ≥1000 bp) but their proportion cannot exceed 10%. The kmer size was kept to the default value (k = 4) for all binners except for mycc since, as suggested by the authors (32), we obtained the best results with 5p6mer (pentanucleotide and palindromic hexanucleotide)

Cat.	Binner	Version	Abundance data	Composition data	Gene length filter	Run information	Reference
Ab.	mspminer	Last update: 25 April 2018	Raw read counts	–	–	–	(21)
Ab.	mgs-canopy	Last update: 9 November 2015	Gene length normalized base counts	–	–	Final bins >700 genes	(20)
Hyb.	solidbin	1.0 (python)	Gene length normalized base counts	Gene catalog + precomputed kmer frequencies	500 and 1000 bp (filtered input files)	Mode: SolidBin-naïve	(27)
Hyb.	cocacola	Last update: February 2019 (python)	Gene length normalized base counts	Gene catalog + precomputed kmer frequencies	500 and 1000 bp (filtered input files)	Mode: without alignment or linkage	(28)
Hyb.	concoct	1.0.0	Gene length normalized base counts	Gene catalog	500 and 1000 bp	–	(29)
Hyb.	maxbin	2.2.5	Gene length normalized base counts	Gene catalog	500 and 1000 bp	–	(31)
Hyb.	metabat	2.12.1	Depth file computed from BAM files^a^	Gene catalog	1500 bp	–	(30)
Hyb.	mycc	Last update: 1 March 2017	Depth file computed from BAM files^a^	Gene catalog	500 and 1000 bp	Kmer size: 5p6mer	(32)
Int.	das tool	1.1.1	–	Gene catalog	–	–	(33)

^a^The computation was performed by the jgi_summarize_bam_contig_depth script of metabat and non-necessary columns were removed for mycc.

### Creation of the simulated non-redundant gene catalog

**Figure 1.**
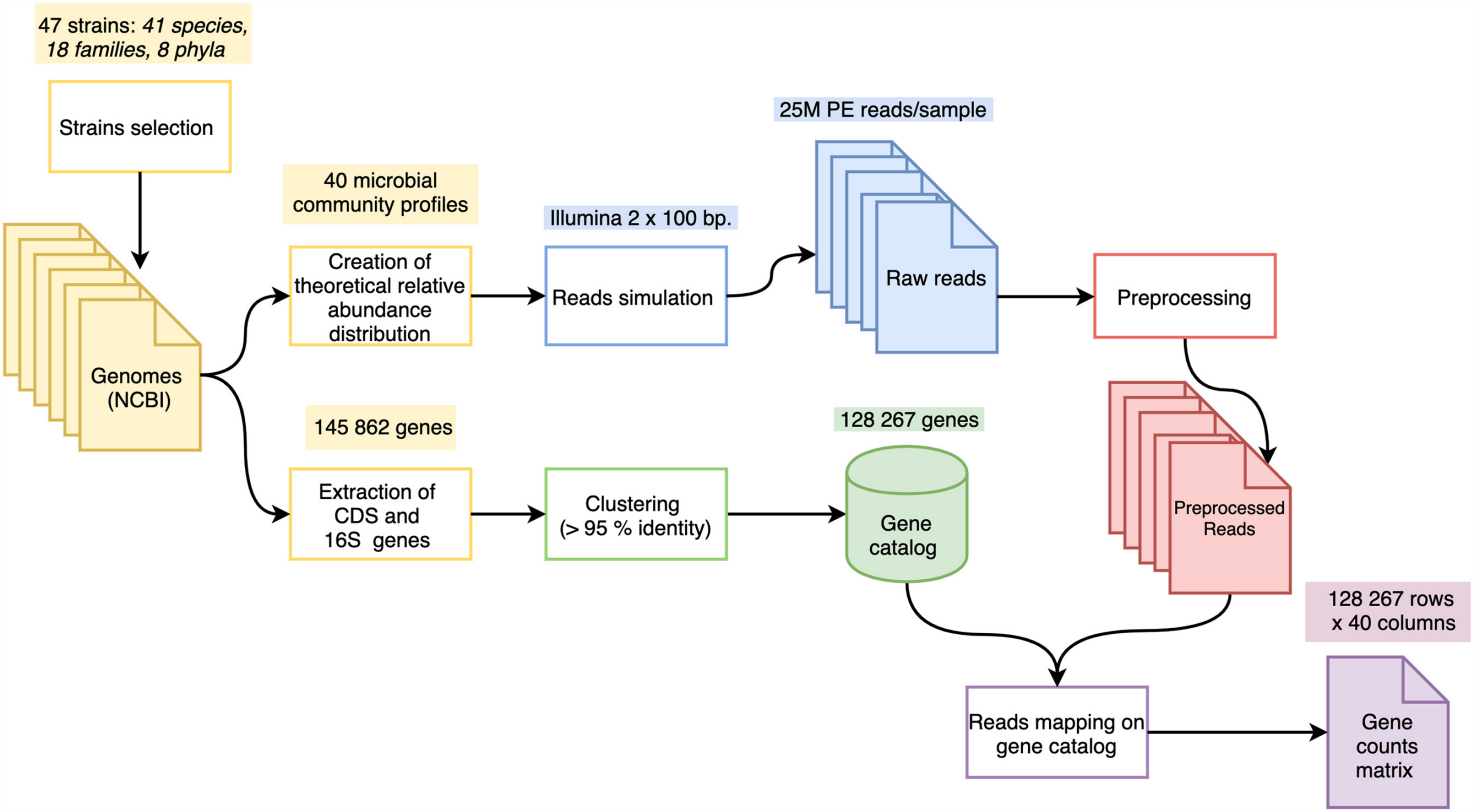
Construction overview of the simulated gene catalog. Colors correspond to the different steps of the workflow: design of the 40 samples and of the gene catalog (yellow), creation of the non-redundant gene catalog (green), simulation of WMS reads for all the samples (blue), reads preprocessing (red) and reads mapping on the gene catalog (purple).

In order to create a simulated human gut microbiota non-redundant gene catalog (SGC), we designed the workflow illustrated in Figure 1 based on the IGC construction. The steps of our workflow are detailed in the sections below. It should be noted that these steps are not fully automated; hence, it has required not only individual in-house scripts but also the manual launching of tools and use of command lines.

#### Strain selection

We designed a SGC composed of 47 strains belonging to 41 microbial species, 40 of which are associated with the human gut microbiota and 26 are part of the ‘core microbiota’. The latter corresponds to a set of bacterial taxa shared by a majority of the analyzed human gut microbiota samples from healthy individuals (2,34,35). Moreover, the strain selection was performed in order to be representative of the taxonomic diversity of the microbiota (Bacteria, Archaea, Virus and Fungi), as well as of several ranges of number of genes, gene length, GC content, codon usage and of elements acquired during genome evolution such as plasmids and prophages (36). Indeed, a bacterial or archaeal strain can acquire mobile elements through different integration mechanisms such as Horizontal Gene Transfer (HGT) or bacteriophage infection (37). These elements can confer selective advantages such as antibiotic resistance (36,38) or metabolic capabilities (37,39). We focused our interest on plasmid and prophage genes that are both expected to have a distinct composition from their host strain (depending on how long they have been incorporated in the host cell). Closely related genomes (40) were also added by identifying known strains with a tetranucleotide frequency correlation (TETRA) above 0.99 and an Average Nucleotide Identity (ANI) higher than 95–96% with jspecies (41). A description of the 47 strains included in the SGC and their taxonomy, as well as a representation of the corresponding taxonomic tree are available in Supplementary Tables S1, S2 and Figure S1.

#### DNA, RNA sequences and non-redundant gene catalog

For each genome (contigs or complete sequence), we retrieved the NCBI ftp links for the genomic, coding and RNA sequences using the R package reutils (version 0.2.3, Gerhard Schöfl 2016) based on the RefSeq or GenBank accession number and we downloaded the corresponding files. We then concatenated the 16S rDNA sequences with the coding sequences (CDS) from all genomes to obtain a single FASTA file. The resulting 145 862 genes were grouped into 128 267 clusters with cd-hit version 4.6 (42) using a sequence identity threshold of 0.95, and an alignment coverage for the shorter sequence of 0.90. We included 16S rRNA genes since they are present in all bacteria and archaea, and they are known to have a high sequence similarity with each other.

#### Reads simulation, pre-processing and mapping

We manually created theoretical abundance range distributions for 40 samples. Our goal was to represent different microbial community structures such as several samples from one individual going from a healthy (balanced composition) to a dysbiotic state (e.g. decrease in diversity, dominance of one species), different abundance ranges for identical communities, or a same abundance range for distinct communities. The samples differ also in their strain-level presence-absence profiles and in their diversity. A strain is considered present in a sample if its abundance value is not zero, and detectable if it has a minimum abundance of 0.1%, approximating a 2× coverage. As for the alpha-diversity of the samples, their richness ranges between 4 and 41 species, and their Inverse Simpson index from 1.53 to 29.50. The 40 theoretical abundance profiles along with representations of the taxonomy and diversity of the simulated samples can be found in Supplementary Table S3 and Figure S2. Based on these distributions and the downloaded genomic sequences, we simulated 25 million paired-end reads per sample (2 × 100 bp, Illumina) using gemsim version 1.6 (43) with the ‘ill100v5_p’ error model. In all the samples, each plasmid of a given strain was simulated in single copy. The following pre-processing steps were performed on the generated FASTQ files: removal of N-containing sequences using vsearch version 2.8.0 (44), removal of single reads (using the python script fastqCombinePairedEnd available at https://github.com/enormandeau/Scripts), quality trimming and filtering using trimmomatic version 0.36 (45). We used mocat version 2.0 (46) to map reads with soapaligner2 (47) using the ‘allbest’ mapping mode and to calculate the gene abundance profiles across samples.

### Benchmarking workflow

#### Input data and parameters setting

Each binner was benchmarked on the SGC using gene abundance profiles and/or composition information but required different input types and formats (Table 1 and Supplementary Figure S3). When necessary, we used the python script reorder_fasta (available at https://github.com/JLSteenwyk/Molecular_sequence_analyses_scripts) to sort the genes of the SGC according to the gene abundance profiles and/or composition information. The binners were launched with default parameters with the exception of the settings described in Table 1. For hybrid binners, we essentially adjusted the minimum gene length filter. Indeed, most of these binners recommend discarding short sequences (generally below 1000 bp) to avoid skewed tetranucleotide frequencies (48). Moreover, we only present the output of das tool based on the binning results of abundance-based binners since the results of das tool were identical when including the binning results of hybrids binners.

#### Output data

We converted the obtained binning results to the Bioboxes format used in CAMI and amber for the benchmarking of binners. We used the python script convert_fasta_bins_to_biobox_format proposed by amber v1.0.3 to convert the output bins stored in a FASTA format, and an in-house R script and bash commands to convert other output formats (e.g. csv or bin-specific files). For mspminer, we included all categories of genes (core, shared and accessory) without distinction for each bin. The final set of bins for each binner was used as output with the exception of mgs-canopy for which we kept only bins >700 genes to conform to the definition of an MGS (20). Each formatted output was then also converted using an in-house R script to a Cluster Node List (CNL) format in order to compute metrics comparing covers. Each line corresponds to a bin and is composed of tab-separated numerical values representing genes; these values range from one to the total number of binned genes. We represented each unassigned gene by a singleton bin to prevent potential biases in the paired comparison of the binning results due to unsynchronized gene sets (i.e. a gene assigned by one binner and not by the other).

#### Application to the integrated gene catalog

We applied the best-performing binner of each category (mspminer, maxbin, das tool) to the IGC and compared the obtained binning results with the ones obtained on our SGC. To do so, we downloaded the gene sequences (IGC.fa.gz) along with the abundance profiles table for 1267 samples (1267sample.gene.pairNum.table.gz) published by Li *et al.* (49). Depending on the binner, we have normalized and formatted the abundance profiles in the same manner as for the SGC (as described in the section on input data), with the exception of maxbin for which base counts were used for the SGC instead of the provided insert counts for the IGC. Moreover, since das tool was used on the output of mspminer and mgs-canopy, we launched mgs-canopy on the IGC. To this end, we rarefied and normalized the counts by gene length for both maxbin and mgs-canopy. The rarefaction was not performed on the SGC given that we simulated the same number of reads per sample. For maxbin, we also had to split the final abundance profile table into sample-specific files structured with two tab-separated columns, corresponding to the gene identifier and the gene abundance in a given sample. Considering the consequent size of the abundance input table, we used the C++ version of rtk (50) to rarefy the counts by setting the depth threshold (i.e. maximum number of sequences per sample) to 11 million reads. This same threshold was used by Nielsen *et al.* (20) and allowed to retain most of the samples (only 122 removed). It should also be noted that we did not run mspminer since the results of this binner on the IGC were already published (21).

### Quality assessment of bins

#### Implementation

We created a fork of the GitHub repository of the quality assessment tool amber v1.0.3 to allow the evaluation of binners on a non-redundant gene set. Overall, the following features were added: assignment of sequences to more than one bin (multi-assignment), computation of metrics based on the number of sequences instead of their cumulated length, adjustment of the implementation of the percentage of assigned sequences and of the number of high-quality (HQ) bins to take multi-assignment into account, computation of the Generalized Normalized Mutual Information (GNMI) (51) to replace the Adjusted Rand Index (ARI) and generation of interactive heatmaps to facilitate the comparison of gene assignments with the expected set of bins (gold standard).

#### Gold standard

The non-redundant gene catalog was converted to the Bioboxes format. To do so, we retrieved the species assignment of each representative gene and of all the genes of the corresponding cluster and associated these species with the identifier of the representative gene. This means that if a gene belonging to the species A is the representative gene of a cluster including genes belonging to the species B and C, it will be assigned to the species A, B and C. The gold standard file consists of a two-column tab-separated text-file (gene identifier and species name) composed of as many lines as the number of unique complete assignments (i.e. if two genes from the same species are clustered together, the representative gene of this cluster will be assigned only once to this species). Supplementary Table S4 summarizes the number of genes and assignments at each step of the creation of the gold standard. The gold standard file was then converted to a CNL file to compute metrics comparing covers. This gold standard corresponds to the expected species-level bin set with which we compared the predicted bin set obtained with each binner. It should be noted that we also created a gold standard including only a single assignment (SA) per gene in order to propose a comparison with the gold standard used to evaluate the methods.

#### Metrics

Based on the definitions provided by amber, we present below the quality assessment metrics and approaches used for the benchmarking and how we extended them to covers. Briefly, the approach of amber v1.0.3 consists in identifying for each predicted bin, a ‘best mapping’ expected bin either based on their shared number or fraction of base pairs. Two main metrics are then computed between each predicted bin and its best mapping expected bin: the ‘completeness’ (i.e. sensitivity) and the ‘purity’ (i.e. specificity). Prior to computing these two metrics, we revised the approach to map a predicted bin (benchmarked binner) to an expected bin (gold standard). With the aim to assess how well and how many genes of the catalog are clustered independently of their length, we consider as best mapping the expected bin sharing the largest proportion of its genes with the predicted bin. If more than one expected bin corresponds to this definition, the one with the largest number of genes is selected. Apart from bin mapping and considering the number of genes instead of the number of base pairs, the computation of purity, completeness, average purity and average completeness remains unchanged as shown in Table 2. As mentioned in the paper (26), average completeness is computed on all the predicted bins along with the unassigned expected bins associated with a completeness of zero.

**Table 2.** Quality assessment metrics. The set of predicted bins and expected bins (gold standard) are, respectively, represented by $X$ and $Y$. Each predicted bin $x \in X$ is assigned to its best mapping expected bin $y \in Y$ before computing purity, contamination and completeness. $| x |$ equals to the number of genes in the predicted bin $x$ and ${Y_{unassigned}}$ represents the set of expected bins that do not correspond to the best mapping of any predicted bin and are associated with a completeness of 0. NMI corresponds to the Mutual Information (MI) normalized by the maximum of the unconditional entropies of the expected and predicted bins $H( Y )$ and $H( X ).$$I( {Y:X} )$ represents the definition of MI where the normalized number of shared genes between a given predicted bin $x$ and an expected bin $y$ is defined as $m( {y,x} )$; and the normalized number of genes in $x$ and in $y$ are $m( x )$ and $m( y )$.

Metric	Formula	Reference
Best mapping expected bin	$bmap\ ( x ) = {}_{y \in Y}^{argmax}{\mathrm{\ }}\ \frac{{| {x \cap y} |}}{{| y |}}$	(26)
Purity	${p_x} = \frac{{| {x\,\, \cap \,\,bmap( x )} |}}{{| x |}}$	(26)
Contamination	${c_x} = {\mathrm{\ }}1 - {p_x}$	(26)
Completeness	${r_x} = \frac{{| {x\,\, \cap \,\,bmap( x )} |}}{{| {bmap( x )} |}}$	(26)
Average purity	$\bar{p} = \frac{1}{{| X |}}\ \mathop \sum \limits_{x \in X} {p_x}$	(26)
Average completeness	$\bar{r} = \frac{1}{{| X | + | {{Y_{unassigned}}} |}}\ \mathop \sum \limits_{x \in X} {r_x}$	(26)
High-Quality (HQ) bins	${c_x} < {0.05\,\,\,\& \,\,{r_x}} >0.90$	(52)
Normalized Mutual Information (NMI)	${NMI\ }( {{Y},{X}} ) = \frac{{{I}( {{Y}:{X}} )}}{{max( {{H}( {Y} ),{H}( {X} )} )}}$ , with ${I\ }( {{Y}:{X}} ) = \mathop \sum \limits_{{y} \in {Y}} \mathop \sum \limits_{{x} \in {X}} m( {{y},{x}} ){{\ log}_2}\frac{{{m}( {{y},{x}} )}}{{{m}( {y} ) {m}( {x} )}}$, and ${H\ }( {X} ) = {\mathrm{\ }} - \sum\limits_{{x} \in {X}} {{m}( {x} )\,\,{{log}_2}{ m}( {x} )}$	(51)

Given that a gene can be assigned to more than one bin, we replaced the ARI by the GNMI as implemented by Lutov *et al.* (51). While the ARI computes the similarity between two partitions (predicted bin and best mapping expected bin), the GNMI corresponds to an extension to covers of the Normalized Mutual Information (NMI). This measure is compatible with conventional NMI values and focuses on the number of common sequences for each pair of clusters between two covers. We also revised the computation of the ‘proportion of binned genes’ and of ‘HQ bins’ to avoid a potential bias due to multi-assigned genes. A gene is considered binned if it is assigned to ‘at least one’ predicted bin. As for HQ bins, they correspond to the set of predicted bins with >90% completeness and <5% contamination (52). However, if several predicted bins are mapped to the same expected bin (best mapping), the count of the HQ bins is only incremented by one. This case should be distinguished from the ‘number of bins’ also presented in our results, corresponding to raw values and where two predicted bins can be mapped to the same expected bin (species).

Moreover, the best mapping of a predicted bin differs from the species it ‘represents’ (one or several) that we use to estimate the predicted bin counts per species. Based on the proportion of a core minimal bacterial gene set composed of 206 genes (53), on an average bacterial genome size of 5 Mb (54), and an average bacterial gene length of 1000 bp (55), we considered a predicted bin to be representative of a species if it shares at least 4% of the genes of the species-level expected bin. Finally, in order to evaluate the assignment of 16S rRNA, selected plasmid and prophage genes, we classified them into three groups: (i) incorrectly assigned (i.e. a gene is not found in any of the representative predicted bins of the expected species); (ii) partially correctly assigned (i.e. a gene is found ‘in at least one’ representative predicted bin of ‘one’ expected species); and (iii) correctly assigned (i.e. a gene is found in ‘at least one’ representative predicted bin of ‘each’ expected species). Considering our initial gene clustering step to create the SGC, it should also be noted that a 16S rRNA gene can be shared by up to three strains, whereas except for 1 prophage gene shared among the strains of *Bifidobacterium longum*, all selected plasmid and prophage genes are specific to one strain present in the SGC. More information on the genes included in this evaluation is available in the Supplementary Data.

## RESULTS

In order to assess the suitability of different binning methods to cluster a large non-redundant set of genes such as the IGC into species-level bins, we selected and applied to our SGC nine taxonomy-independent binners implementing abundance-based (mgs-canopy and mspminer), hybrid (solidbin, cocacola, concoct, metabat, maxbin and mycc) and integrative (das tool) approaches. We present the results of our comprehensive evaluation comprising on one hand overall benchmarking results using a gold standard and metrics taking into account the assignment of genes to multiple bins, and on the other hand a deeper exploration of the results of the best-performing binners based on specific definitions, representations and points of comparison allowing to exploit the particularities of our SGC.

### Overall benchmarking results on the simulated non-redundant gene catalog

#### Percentage of binned genes

**Figure 2.**
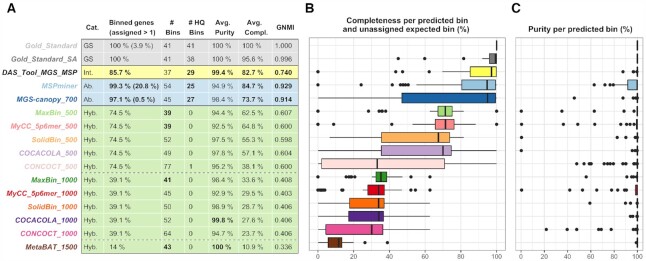
Overall results of the quality assessment metrics for each binner and used parameters. Panel (**A**) shows the computed quality assessment metrics. Binners are grouped and colored by category: gold standard (GS, gray), integrative (Int., yellow), abundance-based (Ab., blue), hybrid (Hyb., green). Continuous lines separate the categories and dashed lines divide the hybrid binners according to their value for the gene length filter (500, 1000 and 1500 bp). For each metric, the top three values are highlighted in bold. Zeros are not represented in the table for genes assigned more than once. Columns representing the number of bins and the number of HQ bins should be read together given that the number of bins does not necessarily represent distinct species. The GNMI score was computed between each binner and the gold standard. Panels (**B** and **C**) show the distribution of the completeness and the purity per bin. Two shades of the same color were used to represent a same binner with a different gene length threshold (e.g. green for maxbin). On panel (**B**), unassigned expected species-level bins are represented by a completeness of zero.

The proportion of genes assigned to a bin varies from 14% up to 99.3% (Figure 2). The highest proportion is reached by mspminer followed by mgs-canopy and das tool (97.1 and 85.7%). Most of the unassigned genes are due to filtering criteria. A threshold on gene minimum coverage and prevalence, and one on the minimum bin size are applied by the first two methods. As for das tool, only genes assigned to bins passing a score threshold computed based on Single-Copy Marker Genes (SCMG) are selected. Hybrid binners tend to bin less genes since they discard short sequences to avoid skewed tetranucleotide frequencies. Except for metabat which binned 85.8% of the filtered genes, the proportion of binned genes matches exactly the number of genes meeting the gene length threshold (74.5% ≥500 bp, 39.1% ≥1000 bp, 16.4% ≥1500 bp). Moreover, as in our gold standard with SA, hybrid methods assign a gene to a single bin only, whereas abundance-based methods assign up to 20.8% (mspminer) of the genes more than once. Compared to our gold standard (3.9% of genes are assigned to more than one bin), mspminer tends to overestimate the number of genes shared between bins (20.8%) while mgs-canopy underestimates it (0.5%). Nonetheless, the integrative approach das tool strictly assigns genes to a single bin and does not group as many genes (85.7%) as its input binning results (mgs-canopy and mspminer).

#### Purity and completeness per bin

While all binners have an average purity above 92%, abundance-based and integrative binners show a higher average completeness (Figure 2). For hybrid binners, we observe a progressively increasing shift in the distribution of the completeness per predicted bin along with the proportion of binned genes, when varying the gene length threshold from 1500 to 500 bp. However, only two of the methods launched with this threshold set to 500 bp have an average completeness value >62% (maxbin and mycc). As represented in Figure 2, both have only a few bins with a completeness below 65% whereas when using the default 1000 bp threshold, most of the bins have a value below 37%. In contrast with completeness values and apart from concoct, we observe a slight decrease in average purity for the methods launched with the 500 bp gene length threshold. This imbalance reaches its extreme with metabat, associated with the highest average purity value (100%) and also to the lowest average completeness value (10.9%). A majority of its bins have a completeness of 13% or less due to an insufficient number of binned genes. Among abundance-based methods, the highest average purity is achieved by mgs-canopy whereas in this category, mspminer has the best distribution and average value for completeness. As expected, the best trade-off between purity and completeness results is obtained by das tool, the integrative approach, based on the binning results of mgs-canopy and mspminer.

#### Number of bins and HQ bins

Globally, the number of bins is well determined by all methods except concoct (Figure 2). It should however be noted that a very good estimation of the number of bins is not sufficient to conclude that all expected species-level bins are recovered by a binner. The top three values are obtained with hybrid methods (39 for maxbin and mycc, 43 for metabat) implementing different approaches for the bin number estimation. The approach of metabat stands out the most by using a graph-based structure (30). Even though mycc and maxbin are both based on the identification of SCMG, they use two different sets of markers and different tools. A similar number of bins is determined by cocacola and solidbin. They both start by initializing such number using SCMG and then compute a silhouette coefficient (56) to select the final number. The gene length threshold affects the final number of bins in different ways depending on the methods. solidbin and concoct output more bins using the 500 bp threshold whereas cocacola, mycc and maxbin output more bins using the 1000 bp threshold. For maxbin, its bin number estimation even results in the exact expected value (41 expected species-level bins). Relying on a variational Bayesian approach to determine the number of bins (29), concoct shows the highest number of bins that is also the furthest from the expected value; however, it is the only hybrid method to recover at least one HQ bin. This bin shares 100% of its genes with the expected bin representing the species *Rotavirus A*. Having several genes with a length below 1000 bp, this expected HQ bin is only recovered when setting the threshold to 500 bp. Interestingly, none of the other hybrid binners is able to recover this bin with a high-quality (>0.90 completeness, <0.05 contamination). solidbin mixes the genes of *Rotavirus A* and of *Candida albicans* and splits them between two bins. cocacola, maxbin and mycc output a bin containing all of the *Rotavirus A* genes combined with either 88% of the genes of *C. albicans* or 68% of the genes of *Dorea formicigenerans*. Predictably, the three methods without a gene length filter (abundance-based and integrative) recover a higher number of HQ bins.

Comparing the abundance-based methods, mgs-canopy recovers more HQ bins than mspminer while generating less bins. This results in a number of bins closer to the expected value for mgs-canopy. Nevertheless, only bins with >700 genes were kept in the case of mgs-canopy, retaining less than half of the original number of bins. By selecting only high-score bins and ensuring that genes are not shared between bins, das tool manages to recover a higher number of HQ bins than both of its input binning results (mspminer and mgs-canopy), which represents 71% of the total number of expected species-level bins. As shown by the comparison of the number of HQ bins between the gold standard and the gold standard SA, binners proposing only a single assignment cannot reach the expected number of HQ bins since to reach a full completeness, several bins are required to share genes.

#### Similarity of the obtained binning results

We used the GNMI metric to compare the sets of expected (gold standard) and predicted bins for each benchmarked method. The resulting values displayed in Figure 2 can be grouped in five distinct ranges, ordered by decreasing similarity: (i) >0.900 for abundance-based methods; (ii) 0.740 for the integrative approach; (iii) >0.590 and <0.700 for hybrid methods with a 500 bp gene length threshold; (iv) >0.400 and <0.500 for hybrid methods with a 1000 bp threshold; (v) 0.336 for the hybrid method with a 1500 bp threshold. Surprisingly, das tool is associated with a lower GNMI value than the two abundance-based methods even though it reaches the best trade-off between the average completeness and purity per bin.

**Figure 3.**
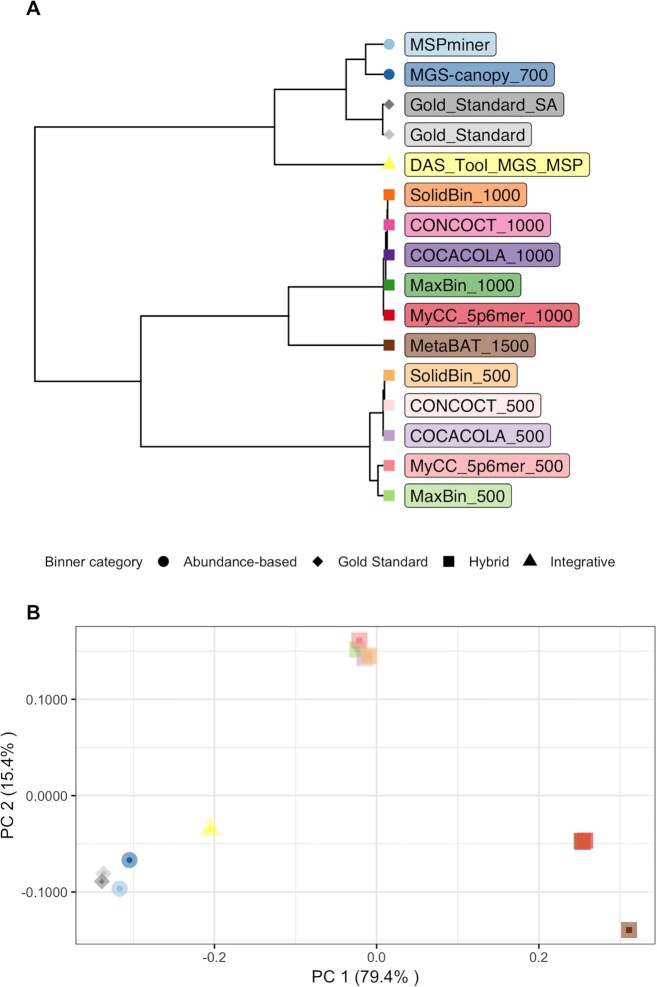
Representation of the GNMI similarity metric between each pair of binners using a dendrogram and a principal coordinates analysis. Binners are represented by different shapes (binning category) and colors. Two shades of the same color were used to represent a given binner with a different gene length threshold (e.g. green for maxbin). Panel (**A**) is a dendrogram representation of the hierarchical clustering results based on GNMI values between pair of binners. These values were also used as input of a principal coordinates analysis which results are represented in panel (**B**).

We also computed the GNMI similarity index for each pair of binners and performed a hierarchical clustering and a principal coordinates analysis on these values. The results of both analyses are displayed in Figure 3. In accordance with the overall binning results, abundance-based and integrative binners are closer to our gold standard than hybrid methods. Interestingly, mgs-canopy and mspminer are closer to each other than to das tool, representing partial results of both individual methods. As for hybrid binners, three groups of overlapping points depicting the three gene length thresholds are distinguished. Considering these observations, we selected the following methods to further explore their results in the next sections: das tool, mspminer and maxbin_500. Each of these binners has reached the highest GNMI similarity value with the gold standard in their respective binning category (Figures 2A and 3B). Moreover, as indicated in the Supplementary Data, these binners also had reasonable computational requirements on our SGC.

### Comparison with the expected community structure at the species and strain levels

#### Global gene assignment profiles

**Figure 4.**
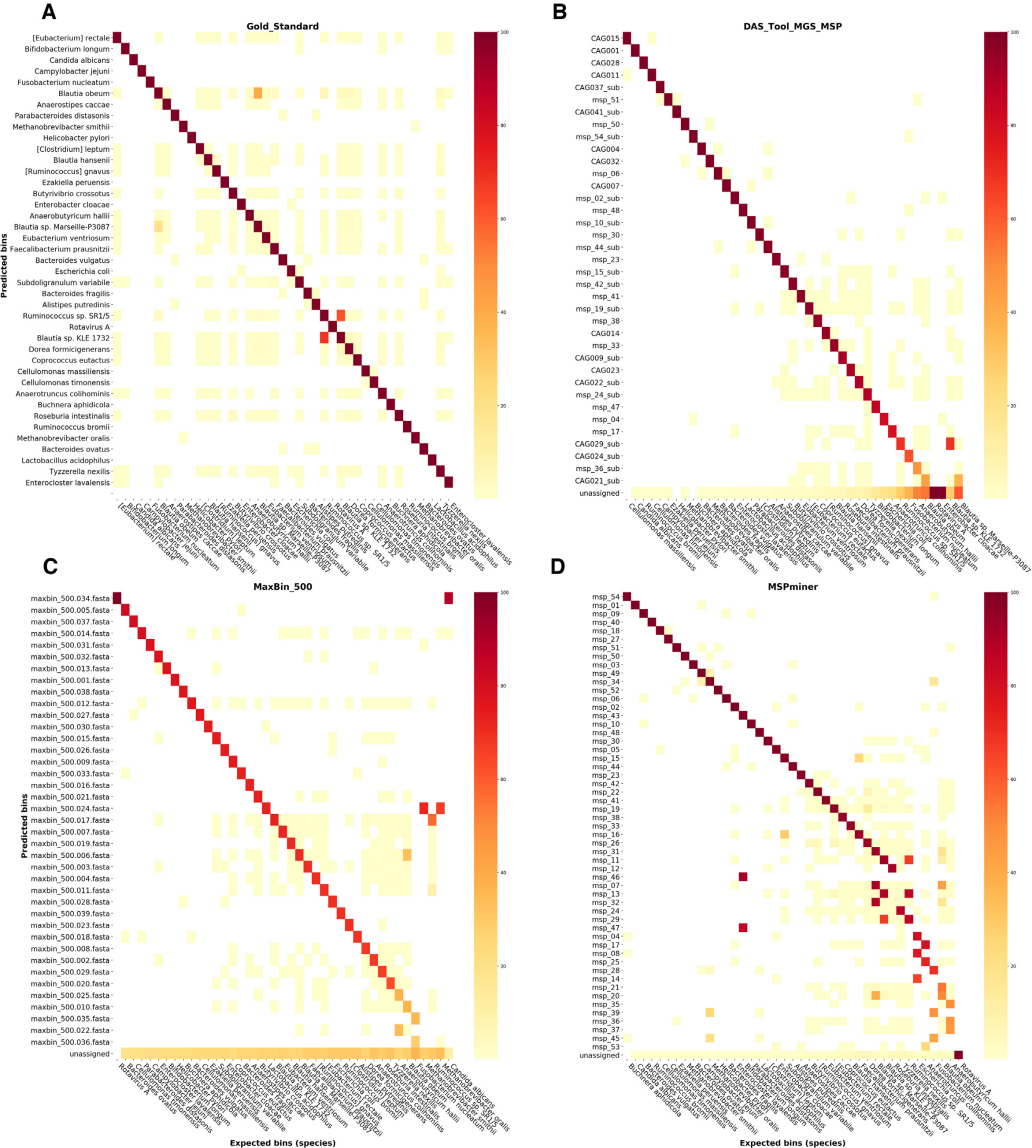
Heatmaps representing the proportion of shared genes between the set of bins predicted by each selected binner and the set of expected species-level bins constituting the gold standard. Each row represents a predicted bin and the last row is dedicated to the genes unassigned by the binner. Each column represents an expected bin (41 species). Each squared dot is colored according to the following proportion: number of shared genes between a predicted bin (row) and an expected bin (species, column) divided by the total number of expected genes in the corresponding species. Rows and columns are sorted by decreasing proportion of shared genes. The panel (**A**) represents a heatmap of the gold standard results for which the set of expected species-level bins was compared with itself. The pair of predicted/expected bins identified by the diagonal line are indicative of the best mapping expected bin for each predicted bin. The heatmaps in panels (**B**–**D**) correspond to the results of the three selected best performing binner of each category: das tool, maxbin and mspminer.

Figure 4 illustrates the number of shared genes between each of the selected sets of predicted bins and our set of species-level expected bins (gold standard) divided by the expected total number of non-redundant genes in each species. As indicated by the continuous red diagonal line in Figure 4A representing the comparison of our set of species-level expected bins with itself, each expected bin shares 100% of its genes with only one predicted bin. Considering that we evaluate binners on a non-redundant gene set, a small (e.g. *Subdoligranulum variabile* and *Faecalibacterium prausnitzii*) and a large proportion of genes (e.g. *Blautia* sp. Marseille-P3087 and *Blautia obeum*) are also shared between multiple bins. Therefore, in contrast with benchmarking results focusing on the reconstruction of individual genomes from contigs, the heatmap representation of the gold standard results also contains scattered light-colored squared dots and few darker ones not positioned on the diagonal line.

Overall, heatmaps of the three selected binners are distinguished by singular profiles. We observe an almost perfect diagonal line for the results of das tool and maxbin (Figure 4B and C), whereas the heatmap displaying the results of mspminer contains a diagonal line with a fractured end (Figure 4D). This indicates that several expected bins share a large proportion of genes with multiple predicted bins (e.g. *Escherichia coli*). A secondary line at the bottom of the heatmap representing the results of maxbin (Figure 4C) corresponds to the proportion of unassigned genes per expected bin. Except for *Rotavirus A* and *C. albicans*, this proportion ranges from 18% (*Bacteroides ovatus*) up to 36% (*Fusobacterium nucleatum*). This relates to the fact that hybrid binners with a gene length threshold set to 500 bp are only able to assign 75% of the genes from our SGC (Figure 2). Additionally, this line shows that the proportion of unassigned genes is evenly distributed between Archaea and Bacteria species-level bins whereas it is smaller for the viral and eukaryote species present in our SGC.

**Figure 5.**
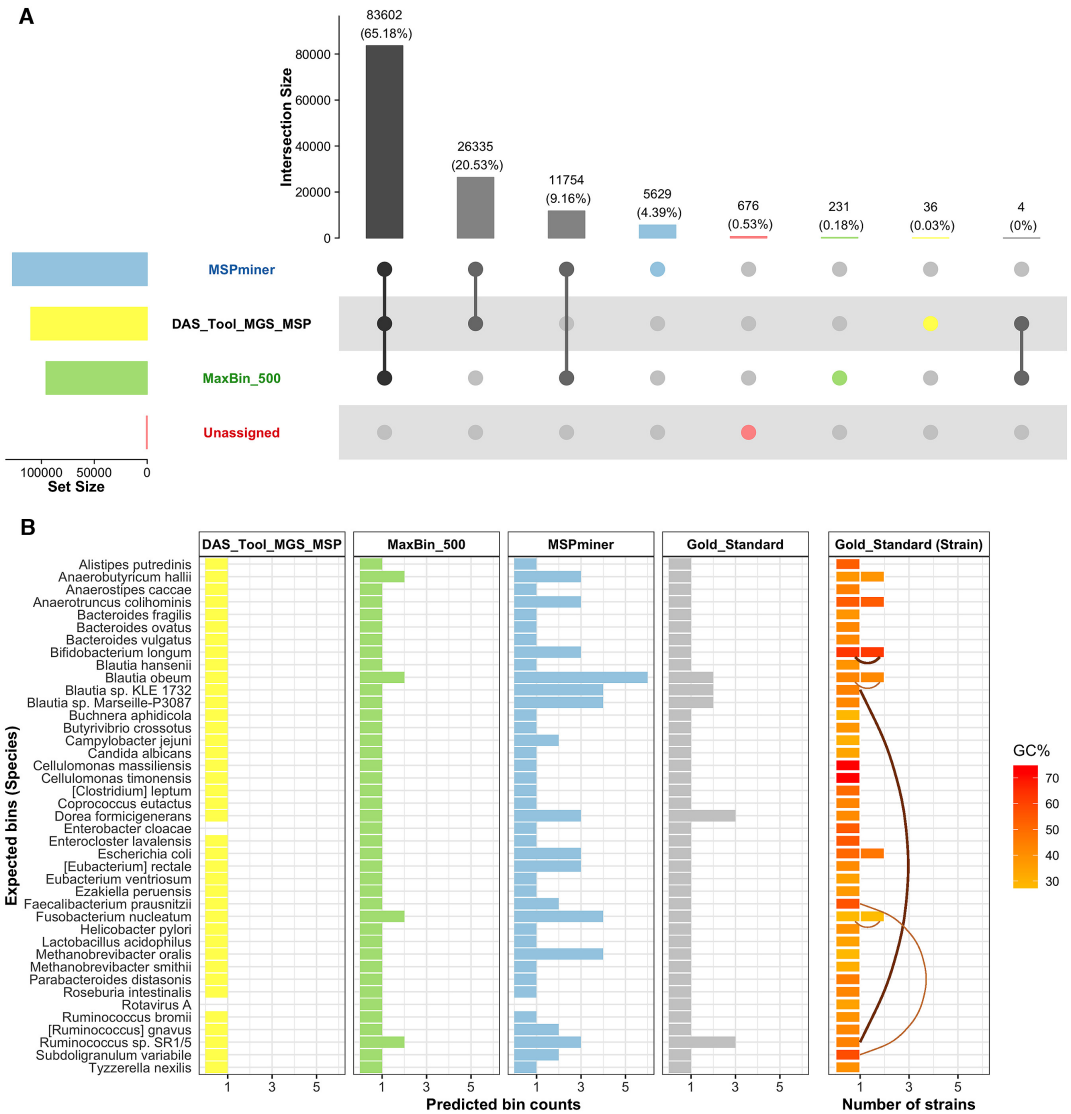
Percentage of binned genes and predicted bin counts per species for the best-performing binner of each category. The comparison of gene assignments between the three selected binners is represented as an UpSet plot on panel (**A**). The values correspond either to the number or the proportion of assigned genes (100% being the 128 267 genes constituting the SGC). Each column represents an intersection and the sets that are part of it are indicated by a filled circle. The size of the intersections is shown in the top bar chart and the size of the sets in the left bar chart. Panel (**B**) represents for each expected species-level bin, the number of representative predicted bins recovered by the three selected binners and by our gold standard (species). A predicted bin is considered representative of a species if it shares at least 4% of the genes of the species-level expected bin (see ‘Materials and Methods’ for more information). The characteristics of each expected species are represented on the right, including the following information: number of strains (up to two strains for a species), average GC content of the genome of each strain, ANI and TETRA values between some selected genomes (belonging to the same or to a different species). ANI and TETRA values were retrieved prior to the evaluation in order to help selecting closely related genomes. They are represented by curved lines which color and thickness are indicative of the pair of TETRA and ANI values: thick brown lines indicate that both metrics reach the values defining closely related genomes (TETRA >0.99 and ANI >95%); thin light brown lines show that at least one of the metrics does not reach the previous thresholds.

Although their assignment to bins may differ, 94.9% of the genes are assigned by at least two of the selected methods (Figure 5A). As shown in Figure 2, hybrid methods bin less genes from our SGC than abundance-based methods. Nevertheless, Figure 5A demonstrates that maxbin is able to assign 231 genes (corresponding to 0.18% of the total number of genes) assigned neither by mspminer nor by das tool. It should also be noted that this proportion of genes uniquely assigned by maxbin represents 17 793 IGC genes.

#### Representation of expected species-level bins by predicted bins

Figure 5B summarizes the number of predicted bins representative of each expected species-level bins. Among the three selected binners, maxbin is the only one recovering all species whereas mspminer fails to represent *Rotavirus A*. Composed of a small number of genes (11 genes), this expected bin cannot be recovered either by mspminer or by mgs-canopy due to their filter on bin size applied respectively upstream (bin size ≥150 genes) and downstream (bin size >700 genes) of the final bin generation. Consequently, this species-level bin is also absent from the results of das tool, along with the expected bin of *Enterobacter cloacae*. As shown in Figure 5B, this latter bin is well recovered by both mspminer and mgs-canopy (>98% of shared genes). However, none of their respective bins passes the das tool default score threshold of 0.5 due to the identification of less than half of the expected set of SCMG, leading to respective scores of 0.41 and 0.39.

#### Over-representation of species-level bins

As shown in Figure 5B, das tool outputs a maximum of one bin per species, while the other binners represent each species with up to two (maxbin) and six (mspminer) predicted bins. Considering the six species composed of two strains in our SGC, all of them were represented with at least three bins by mspminer, of which three were represented with two bins by maxbin. These latter three species (*B. obeum*, *Anaerobutyricum hallii*, *F. nucleatum*) have a low to medium average GC content, while the ones over-represented by mspminer only (*Anaerotruncus colihominis*, *B. longum*, *E. coli*) have a medium to high average GC content. Among those and considering the strains for which we previously had computed ANI and TETRA values, we observe low to medium ANI and TETRA values for the species with two strains over-represented by both binners (*B. obeum and F. nucleatum*, Figure 5B). In contrast, the strains of *B. longum* share high ANI and TETRA values and this species is over-represented by mspminer only.

While in most of these cases of over-represented species both binners output at least one bin per strain, we observe that two representative bins for a species do not always represent two strains. Indeed, mspminer also outputs bins containing a mixture of genes specific to both strains (e.g. msp_17 for *A. colihominis*). Moreover, a predicted bin is not necessarily representative of a single species in the results of both maxbin and mspminer. This is notably the case for *Blautia* sp. KLE 1732 and *Ruminococcus* sp. SR1/5 (e.g. msp_29 and maxbin_500.017, Figure 4C and D), two strains sharing several genes and high ANI and TETRA values. More details on the species-level bins over-represented by these methods are available in the Supplementary Data. As for the integrative binner, it outputs at most a single bin per species and manages in some cases to resolve the latter issues. For instance, the genes from *S. variabile* and *F. prausnitzii* are mixed into two bins by mspminer (msp_15 and msp_16, Figure 4D) and recovered in separated bins by das tool (msp_15_sub and CAG023, Figure 4B). However, das tool sometimes essentially recovers only one of the strains of a species (*A. hallii*), or selects bins still containing genes from other species (*B. obeum* A2–162 and *Blautia* sp. Marseille-P3087, Figure 4B). This is mainly due to its dependency on the quality of its input binning results.

### Distribution of the annotated genes between the predicted bins

We evaluated the distribution of the 16S rRNA, selected plasmid and prophage genes included in our SGC between the predicted bins and present an overview of the obtained results in this section.

#### Assignment of annotated 16S rRNA genes

A first notable difference across the selected binners is the number of assigned 16S rRNA genes. Although none of the three selected binners is able to assign the whole set of 35 non-redundant 16S rRNA genes, 34 genes are assigned by mspminer and maxbin, and 31 by das tool. While maxbin incorrectly assigns ten 16S rRNA genes to a representative bin of another species more or less distant in the taxonomy (genus to super-kingdom), there is no incorrectly assigned 16S rRNA genes by mspminer based on our definition. Indeed, this binner outputs a correct assignment compensating for a partially incorrect assignment of the same gene (e.g. 16S rRNA gene of *Buchnera aphidicola* assigned to the representative bin of *B. aphidicola* and to two of *E. coli*). Moreover, in the case of a gene shared by several strains, mspminer manages to assign it to at least one of the representative bins and sometimes to all the representative bins of the expected species. However, considering the amount of over-represented species, this sometimes leads to a partially incorrect assignment due to a gene assigned to a bin representing multiple species and including unexpected ones (e.g. 16S rRNA gene of *F. prausnitzii* assigned to two bins, including one representative of *F. prausnitzii* composed of a majority of genes of *S. variabile*). As for maxbin which is limited to single assignments, a correct assignment can be due to a bin representing multiple species (16S rRNA gene shared between *Methanobrevibacter oralis* and *Methanobrevibacter smithii*, maxbin_500.024 on Figure 4C). As for das tool, although it allows, for instance, to disentangle the previous situations of the 16S rRNA gene of *F. prausnitzii* by selecting only one bin, it also assigns less genes and has more partially correct assignments than the two other binners. See the Supplementary Data for more details on the distribution of 16S rRNA genes between the predicted bins.

#### Assignment of annotated elements acquired during genome evolution

Overall, considering plasmid and prophage genes, mspminer assigns all the 88 and 30 selected prophage and plasmid genes whereas maxbin is able to bin all the genes >500 bp (57 plasmid and 19 prophage genes). das tool shows intermediate results by binning 41 plasmid and 26 prophage genes, respectively representing 46.6% and 86.7% of all the selected genes for both categories. Among their assigned genes, maxbin, das tool and mspminer output only correct assignments for plasmid and prophage genes. However, as further described in the Supplementary Data, correct assignments are sometimes also associated to missing or partially incorrect ones due, for example, to several representative bins for a species (e.g. the plasmid genes of *E. coli* are assigned by mspminer to two out of the three representative bins of the species, and das tool selects the msp_04 which does not contain most of them) or to bins representing multiple species (e.g. the prophage genes of *F. nucleatum* are assigned by mspminer to the four representative bins of *F. nucleatum*, including three also representative of *M. oralis)*.

### Application of the best-performing methods to the IGC

#### Number of recovered bins

**Figure 6.**
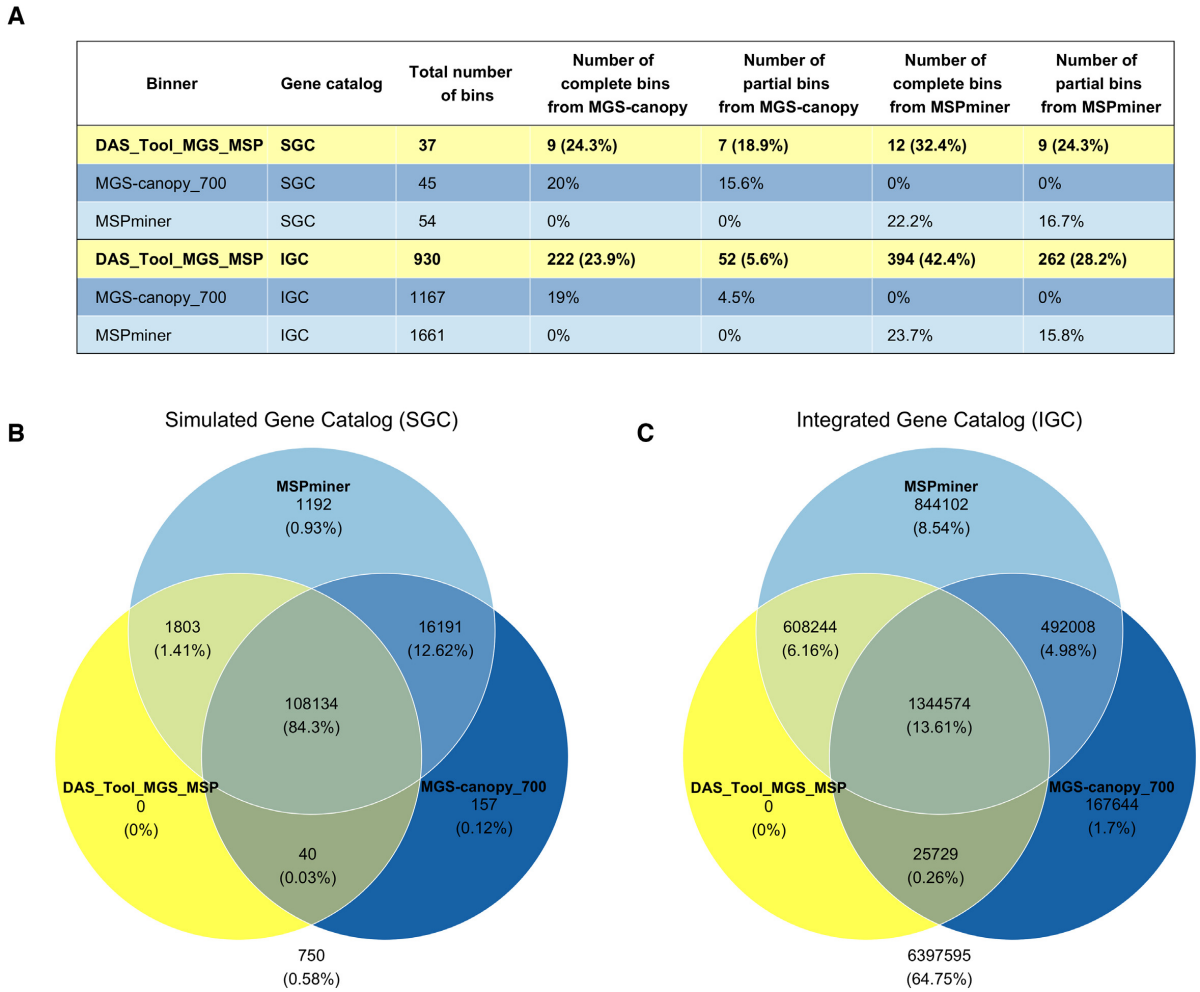
Number of bins and proportion of binned genes between the SGC and the IGC. Panel (**A**) shows the results in terms of bins for the three binners when applied to the SGC and to the IGC. The third column represents the total number of bins recovered by each method and the four columns to its right describe a fraction of these bins. For instance, on the IGC, 222 bins recovered by das tool correspond to complete bins selected from the binning results of mgs-canopy. These 222 bins represent 23.9% of the bins of das tool and 19% of the bins of mgs-canopy. Panels (**B** and **C**) illustrate as Venn diagrams the genes binned by each binner on both catalogs (SGC and IGC). The values correspond either to the number or the proportion of assigned genes (100% being the total number of genes of the SGC or the IGC). The ones not in any circle indicate the unassigned genes.

Figure 6A shows a comparison of the number of bins obtained on the SGC and on the IGC with mspminer, mgs-canopy and das tool. On both catalogs, mspminer outputs the most bins (54 bins on the SGC and 1661 on the IGC), and das tool outputs the least (37 bins on the SGC and 930 bins on the IGC). It should however be noted that rarefied counts were used for mgs-canopy which may have resulted in the loss of low abundance species, hence a slight increase in the number of bins is to be expected with non-rarefied counts. Globally, we also observe similar values for the proportions of bins selected by das tool from each binner. Indeed, between 19 and 24% of the bins of mgs-canopy and of mspminer were selected in integrality (complete bins) by das tool on the SGC and on the IGC. As for the proportion of partial bins, while it remains stable with 16 to 17% of the bins of mspminer partially selected by das tool, the value decreases from 15.6 to 4.5% for the bins of mgs-canopy. In terms of bins recovered by das tool, we observe a slight increase in the proportion of bins that have been selected completely or partially from the binning results of mspminer (from 32.4 to 42.4% for complete bins). As described above, this is compensated by a decrease in the proportion of partial bins selected from the binning results of mgs-canopy (from 18.9 to 5.6%). In the end, the trends appear to be the same for both catalogs when comparing the number of bins recovered by the three binners and the number of bins selected by das tool. As for the evolution of the computational requirements, as detailed in the Supplementary Data, while the relative trends are similar for mspminer and das tool on both gene catalogs, they are opposite for mgs-canopy since it required the least time and memory on the SGC and the most on the IGC.

#### Percentage of binned IGC genes

The proportion of genes assigned by each binner on the SGC and on the IGC are represented as a Venn diagram in Figure 6B and C. Overall, the percentage of unassigned genes obtained on the SGC (0.58%) is not representative of the one resulting from the binning of the genes of the IGC (64.75%). Indeed, the total proportion of assigned genes per binner was overestimated on the SGC. For instance, mspminer binned 99.3% of the genes of the SGC but only 33.3% of the genes of the IGC. Concerning the proportion of binned genes assigned to more than one bin, while it remains stable for mgs-canopy between both catalogs (0.5% on the SGC and on the IGC), it largely decreases for mspminer when applied to the IGC (20.8% on the SGC and 2.1% on the IGC). However, this latter binner remains the one that binned the most genes from both catalogs and das tool is the one that binned the least (85.7% on the SGC and 20% on the IGC). Nevertheless, the proportion of genes assigned only by one binner in the results of das tool is slightly higher in relation to the IGC. It goes from 1.41 to 6.16% at the intersection of mspminer with das tool, and from 0.03 to 0.26% at the intersection of mgs-canopy with das tool. Although these proportions are relatively small, they correspond to a total of several hundred thousand IGC genes (608 244 for mspminer and 25 729 for mgs-canopy). Moreover, around 15% of the genes of the IGC (equivalent to 43% of the binned genes) are assigned by either one or the two abundance-based binners but not by das tool. Therefore, this proportion shows how much room for improvement there is using an integrative approach based on these two binning results.

## DISCUSSION

### Evaluation of binners on a non-redundant gene catalog

Standard metrics (26,52) and test datasets (25) have been proposed to evaluate the recovery of MAGs from the assembled contigs of metagenomes. To our knowledge, there is yet no established consensus on how to compare species-level bins built from a non-redundant set of genes. Moreover, although the CAMI challenge has defined a framework to benchmark metagenomic tools, there are few independent published benchmarking results and still a considerable heterogeneity in the evaluation of binners. Quality assessment results are generally obtained either with commonly used tools based on SCMG (57,58) or by comparing the results to a gold standard using tools like amber, which was developed to compute standard metrics during the CAMI challenge. Nevertheless, even with defined standard quality assessment metrics, benchmarking results are not always comparable. Between the SCMG-based approaches, the implementation of metrics and especially the set of SCMG vary across the studies. As for approaches based on a gold standard, each benchmarking result is specific to the used dataset and in particular to its complexity (e.g. number of sequences, samples, species and strains) and to the microbial ecosystem it represents.

In this study, we proposed a global benchmarking analysis of the binning results obtained from a non-redundant gene catalog with a focus on the human gut microbiota. It should, however, be noted that apart from the design of the SGC (selected microbial species and abundance profiles), our way of comparing the community structure at the species and strain levels along with the distribution of annotated genes (16S rRNA, prophage and plasmid genes) could be applied to non-redundant gene catalogs of other microbial ecosystems. Nevertheless, the design of our SGC is relatively simple and therefore not necessarily representative of the complexity of conventional gut microbial communities. However, it could easily be extended to many other simulated abundance profiles and despite its simplicity, it reproduces most of the essential characteristics of the gut microbiota and remains a good evaluation support for our study. Our SGC could nonetheless be enriched at several levels, for instance by including 23S rRNA genes that are also present in all prokaryotic genomes, chimeras, partial assemblies or also incomplete genes to assess their influence on the binning results given that only 57.7% of the IGC genes correspond to complete open reading frames (8). Furthermore, we have adapted the implementation of standard assessment metrics in amber to also consider covers and have included specific metrics such as the GNMI. We have also proposed a computation that is independent of the sequence length which in our case is necessary given that with a set of non-redundant genes, the aim is not to correctly cluster the largest number of base pairs but the largest number of sequences. As for the extension to covers, while it is important on a non-redundant catalog to take into account the information of genes shared between multiple species, our results remain compatible to partitions if either or both the expected and the predicted bin sets are partitions.

### Complementarity and similarity of the methodological approaches

Although each binner implements a different methodological approach, they are all related within their category either through their required input, part of their main algorithm (e.g. bin number estimation, computed distance and clustering method) or their dependencies. Indeed, all of the selected hybrid binners use kmer frequencies and the majority estimates the number of bins by starting with the identification of SCMG (maxbin, mycc, cocacola, solidbin). Nevertheless, while almost all binners achieve a relatively good estimation, they do not always use the same marker set and neither does das tool that implements an integrative approach using SCMG to compute its scoring function. As for hybrid binners with a methodology independent of SCMG, even though they do not reach high completeness results on our SGC, they have shown good results on other datasets (25,26). Moreover, by considering only 16.4% of the genes of the SGC, metabat outputs a very good bin estimation (43 bins) and most of its bins represent a single species. As for the two abundance-based binners, they rely on the computation of a correlation coefficient on gene abundance profiles across samples, and thereby require a large number of samples.

These methodological resemblances thus lead to similar limitations within the three categories, notably in terms of the used filters. Abundance-based binners essentially apply filters on gene prevalence, gene minimum counts and bin size. Hence, they assign fewer low-abundant genes and only recover relatively large bins. Consequently, they recover less well the species having a small genome (e.g. *Rotavirus A*, mspminer and mgs-canopy). As for hybrid binners, while they are able to recover small bins, they systematically filter genes based on their length, generally removing genes with a length below 1000 bp by default to avoid skewed tetranucleotide frequencies. In our case, this filter leads to the loss of 60.9% of the SGC genes, making it difficult to compare the results of hybrid binners with the output of abundance-based binners. This has led us to try to lower the gene length filter for hybrid binners when it was possible. Nevertheless, we would like to point out that apart from this parameter and the setting of the kmer size for mycc that was advised by the authors, we did not try to optimize the other parameters of each binner. Although it could certainly improve their results, we believe the ones we obtained along with our comprehensive evaluation are enough to make an informed decision on which binners are more adequate to handle specific cases and therefore more suitable to be applied to a given dataset. Besides, our evaluation aims to be representative of the common usage, using either default parameters or setting the main parameters essentially following the recommendations of the authors. Concerning the gene length filter, apart for the main shift, similar relative differences in the distribution of the completeness per bin are observed between hybrid binners when decreasing their gene length filter. Therefore, while taking into consideration the recommendations of the authors, it appears that after properly testing them, some hybrid binners could be used with a lower threshold.

Furthermore, the combination of their results with the output of abundance-based binners, which are not directly influenced by gene length, could help refining the binning results and reducing the impact of potential biases relative to kmer frequencies. In our case, das tool reaches good overall results, but only selects bins passing a defined score threshold. Since the bins obtained by hybrid binners on our SGC are not complete enough to be selected, we were not able to evaluate how das tool could potentially improve the binning results when considering the output of hybrid binners. It should also be noted that, in the IGC, the proportion of genes below the gene length filter (76% <1000 bp) is even higher than on the SGC, and that we also encountered a scalability issue using the best-performing hybrid binner (maxbin). Moreover, since it is based on a set of SCMG from Bacteria and Archaea, das tool is expected to be limited to the recovery of prokaryotic species, even though we encountered an exception (*C. albicans*). Other integrative binners are available but, to our knowledge, they are also based on the computation of completeness and purity using SCMG (59) or essentially aim at refining bins (60). In the end, hybrid and abundance-based approaches have inherent limitations that could be compensated by the use of an integrative binner. This latter approach is very promising but is not yet able to fully exploit the complexity of our set of non-redundant genes.

Counting the genes assigned by at least one of the abundance-based binners, the total proportion of assigned IGC genes reaches 35.25%. This also represents the current maximum potential of das tool on the obtained binning results on the IGC, if we were to fully customize its parameters. We believe better results could be reached on the IGC by combining the complementary results obtained by both abundance-based and hybrid binners. This could be achieved either by including the results of a scalable hybrid method capable to bin short sequences or by an integrative method able to consider bins with a low completeness.

### Different levels of comparison exploiting the characteristics of the SGC

While on our SGC the best trade-off between the completeness and purity per bin was achieved by das tool, its GNMI score with the gold standard was lower than the score of the two abundance-based binners. This suggests that standard quality metrics are not enough to fully characterize and therefore understand the results of the benchmarked binners. Although these computed metrics allow to obtain easily comparable assessment results, they offer only a high-level comparison of binners. For instance, even by adjusting the computation of HQ bins (by counting each expected species once), the number of species represented by each binner remains unclear. This is why we decided to explore in a deeper way the results of the best-performing binner of each category, thereby exploiting the characteristics of our SGC.

To do so, we proposed interactive heatmaps or summary representations (predicted bin counts per species) of the binning results. Based on these representations, we observed that the number of unassigned genes was relatively evenly distributed between species by the hybrid binners (maxbin), that a predicted bin can be representative of multiple species (e.g. *M. oralis* and *M. smithii*, maxbin), that a species can be represented by multiple predicted bins (e.g. *E. coli*, mspminer) and that species composed of a small number of genes are not always recovered (e.g. *Rotavirus A*, mspminer). Finally, considering on one side the large number of binners and their applications, and on the other side, the increasing size and complexity of the gene catalogs, we believe it is critical to characterize as much as possible the obtained results. Therefore, we tried to provide insight into the following questions by analyzing our results at a lower granularity: (i) does a bin represent a species or a strain; (ii) are there expected biases due to composition or taxonomy closeness; (iii) how well are the genes with particular annotations assigned given that, for instance, plasmid genes can have a major impact on health due to the antibiotic resistances they can confer (36,38).

The design of our SGC was intended to include key features that might influence the binning results such as closeness in genome composition (GC content, ANI and TETRA values, shared genes); taxonomy proximity; within-species diversity (one or two strains per species); and gene annotation (16S rRNA, prophage and plasmid genes). To this purpose, we selected various strains in order to represent all these characteristics and adopted a straightforward approach consisting in downloading CDS and 16S rDNA sequences to avoid introducing assembly or gene prediction biases. With the aim to deeply explore the content of the predicted bins, we analyzed the obtained results at the species, strain and gene levels. For the first two levels, we evaluated species representation with each binner, by considering that a predicted bin is representative of a species if it shares at least 4% of the genes of the species. Our first observation was that mspminer over-represented 16 species (accounting for 39% of the total number of species) with up to six bins. maxbin also over-represented four of them with two bins but was the only selected binner able to represent all species with at least one bin. Indeed, two species were unrecovered by at least one of the other selected binners, which can be explained by a bin size filter (*Rotavirus A*, mspminer) and the lack of identified SCMG (*E. cloacae*, mspminer). Finally, while we also observed over-represented species in the gold standard according to our definition, the cause of the over-represented species was not always clear and needed further investigations.

### Impact of the community structure and of genome closeness on the binning of SGC genes

Even though a relation between the taxonomy and the composition of genomes has long been identified and well-characterized with metrics such as the ANI and TETRA, we observed different behaviors between binners. This indicates that between two genomes, composition closeness can be higher or lower than expected by their taxonomy proximity (e.g. within a species, ANI >95% and TETRA >0.99). Based on the results obtained on our SGC, the genome composition appears to strongly impact maxbin. Although its influence is mostly positive (e.g. only a few over-represented species), the composition closeness is not always fully consistent with the expected taxonomy, leading to some cases of bins that each represent multiple species. Moreover, species with high GC content are overall less over-represented, in particular the ones composed of two strains, as compared with the results of mspminer. Indeed, in our results, this method over-represented the six species composed of two strains whereas maxbin was able to assign to a single bin the three pairs of strains which have a medium to high average GC content, and for one pair at least, high ANI and TETRA values. As for das tool, it generated at most one bin per species and allowed to resolve some cases of over-represented species. Nevertheless, for these binners, while some bins represent both strains of a species, others only contain genes from one strain, or a mixture of genes from different species more or less distant in the taxonomy. Besides, for a same species, mspminer sometimes outputs bins representing both strains of a species and also other bins specific to a strain. This is essentially caused by its ability to assign a gene to more than one bin (multi-assignment).

By analyzing the results at the gene level, the impact of multi-assignments, of taxonomy and of shared genes can be better appreciated. For this part, we focused on three types of annotated genes from the SGC: 16S rRNA, prophage and plasmid genes. From our perspective, these genes are of particular interest either due to their function, to the way they were integrated into the genome of the strain, or to their independent mode of replication. Therefore, within each type of annotation, they either have a high sequence similarity between each other or can be found in several copies in one strain. Nevertheless, we did not address this latter aspect in the case of plasmids considering that they were simulated in single copy in our SGC. While the main goal was to evaluate if 16S rRNA, prophage and plasmid genes would be correctly assigned with their expected species by each binner, these different types of gene annotation offer complementary ways to measure the impact of the key features previously defined. Indeed, 31% of the non-redundant 16S rRNA genes are shared by up to three strains whilst most of the selected plasmid and prophage genes are not shared with another strain of the SGC. Thus, the former genes can help emphasize the impact of multi-assignments as compared with single assignments, and the latter the impact of taxonomy without shared genes.

Overall, multi-assignments not only allow to correctly assign the annotated genes shared intra- and inter-species (mspminer), representing therefore a considerable advantage, but can also lead to an over-representation of species, and consequently to a large proportion of unassigned genes by das tool, depending on the bin it selects. We observed also several unassigned, partially correct and incorrect assignments with maxbin either due to the gene length filter, to single assignments or to taxonomy proximity. However, in our SGC, this latter cause seems to essentially influence the assignment of 16S rRNA genes and not of prophage or plasmid genes. Furthermore, it should be noted that we made these observations based on the assumption of a proper assembly of 16S rRNA, prophage and plasmid genes. This is not always the case in the current MAG reconstructions (61) but can be expected in the IGC given that 16S rRNA gene sequences from HMP were included during the construction of this catalog (8).

### Essential consideration for the binning of large non-redundant gene catalogs

In addition to the presented benchmarking results, we have identified the following criteria that are also crucial to consider given the size and applications of the most used (IGC) and of the recently available human gut microbiota non-redundant gene catalogs (Global Microbial Gene Catalog - http://gmgc.embl.de): (i) usability; (ii) scalability in terms of time and memory resources; (iii) reproducibility; (iv) maintenance and comparability of the obtained bins when increasing the size of the reference gene catalog. We believe that these points should be taken into account when selecting or developing a method to bin a large gene catalog whether it is the one of the human gut microbiota or of another microbial ecosystem. It should be noted that we do not provide an exhaustive analysis of all these points but aim instead to highlight their significance using some of the special cases encountered during this evaluation.

We measured the importance of the first three criteria either already on the SGC or when applying the best-performing binners on the IGC. First, we included several aspects under the term usability. The ease of use varied widely between binners, notably in relation to the input format or the limited command-line interface. For instance, some binners do not include the main processing steps such as applying the gene length filter and computing the kmer frequencies (solidbin, cocacola), requiring therefore to perform them manually. Besides, binners were not always as easy-to-use on the IGC as we had observed on the SGC. This was especially the case for maxbin that requires sample-specific gene abundance files as input which took a considerable amount of time to create and of disk storage on the IGC. Furthermore, as previously discussed, the lower boundary on the gene length filter is also a main usability issue, particularly for metabat for which it is set to a value higher than the average prokaryotic gene length. Secondly, time or memory scalability issues were encountered either on the SGC (mycc and solidbin, see Supplementary Data) or when applying the best-performing binners on the IGC (maxbin and mgs-canopy). We noticed that maxbin fixed a hardcoded limit on the number of samples to 1023 that remains acceptable on the IGC but will be a real limitation on larger catalogs. Moreover, we did not obtain any binning results after a week of computation. As for the abundance-based binners, mspminer finishes in a few hours whereas mgs-canopy allows to obtain intermediate results by sending an interruption signal. Thirdly, we believe it is important that binners guarantee the reproducibility of their results either by allowing to set a seed for pseudo random number generation (metabat) or by not requiring a manual interruption. Regarding this latter observation, mgs-canopy also provides an early stopping parameter which can be set instead.

Finally, the last pair of criteria, maintainability and comparability, is all the more relevant considering the potential applications of the results of the evaluated binners in reference-based metagenomic analyses, such as characterizing microbiome samples in the context of research or of clinical studies. As previously mentioned, a first set of species-level bins was established on the MetaHIT catalog, leading us to evaluate the suitability of the available binners to cluster the genes of the IGC. However, the sequences of a larger catalog composed of 52 million non-redundant gut microbial genes have recently become available. Consequently, a different gene catalog, but also species-level bin set were and will certainly be used across the studies and the years. Thus, we believe it is important not only to build a reference set of species-level bins on the most used and on the largest gene catalogs, but also to ensure their continuity. This could be achieved by studies or methods aiming to complement or refine bins recovered from a previous catalog, to improve and make connections with the former taxonomic annotation of a bin (e.g. similar to what is proposed for MAGs in the MGnify website (18,62)), and to follow the evolution of the assignment of each gene. Independently of the selected binner for the initial gene binning, one possibility among others could be to use an integrative approach between the binning results of a previous and of a newer catalog. This could allow to perform a complementary update of the reference set of bins instead of replacing it with a new independent set. Moreover, as recently pointed out for MAGs (63) and although their role is different in metagenomic analyses, the obtained set of bins should be well-characterized, curated and validated.

## CONCLUSION

In conclusion, we have conducted a comprehensive evaluation of nine binning methods at different levels of granularity, taking into account the particularities of the most used gut microbiota non-redundant gene catalog and of its increasing size. To this purpose, we have specifically designed a SGC, created a gold standard composed of genes assigned to more than one bin and adapted the quality assessment tool amber. This allowed us to compare the suitability and usability of binners to recover species-level bins from a non-redundant set of genes. Globally, the three main binning approaches are distinguished not only by specific advantages, but also by inherent limitations. However, among the three selected best-performing binners, only abundance-based or integrative binners were able to cluster the IGC genes. This is mainly because hybrid binners filter sequences based on their length or have scalability issues. Considering the overall low proportion of assigned IGC genes, we believe that mspminer still provides the best available binning results on the IGC, in terms of the proportion of genes assigned by this binner, its scalability and its ability to assign a gene to more than one bin. Nevertheless, although this latter ability is essential, the potential biases it may introduce should be taken into consideration when interpreting the obtained results. Moreover, the benchmarked hybrid and integrative binners show very promising and potentially complementary results but require further improvements in order to make them usable on and scalable to a large non-redundant gene catalog. Throughout our analysis, we further identified important criteria to consider in the selection or development of binners to handle the size and complexity of large non-redundant gene catalogs, which in turn are essential for the characterization and the understanding of the functioning of microbial communities, such as the human gut microbiome. In particular, to ensure the comparability between reference-based metagenomics analyses, we believe it is important to further characterize and to provide a continuity between the binning results obtained on the different versions of the human gut microbiota gene catalogs.

## DATA AVAILABILITY

All mentioned in-house scripts and our adapted version of amber can be found on GitHub (https://github.com/MaaT-Pharma/AMBER). The simulated gene catalog along with the corresponding gene abundance profiles and binning results are available for download on Zenodo (http://doi.org/10.5281/zenodo.4306051).

## Supplementary Material

lqab009_Supplemental_File
