# Supplementary material for: A comprehensive evaluation of binning methods to recover human gut microbial species from a non-redundant reference gene catalog
Source: NAR Genom Bioinform. 2021 Mar 1;3(1):lqab009. doi: 10.1093/nargab/lqab009 (PMC7936653; doi:10.1093/nargab/lqab009)
Supplement: lqab009_Supplemental_File [file lqab009_supplemental_file.pdf]

Supplementary Material for *A comprehensive evaluation of binning methods to recover human gut microbial species from a non-redundant reference gene catalog*

Marianne BORDERES, Cyrielle GASC, Emmanuel PRESTAT, Mariana GALVAO FERRARINI, Susana VINGA, Lilia BOUCINHA and Marie-France SAGOT

SUPPLEMENTARY METHODS 2
Selection criteria for taxonomy-independent binners 2
Computing environment and resources 3
Selection of 16S rRNA, plasmid and prophage genes to evaluate their assignment 3
SUPPLEMENTARY ANALYSES 4
Computational requirements on the SGC 4
Evolution of the computational requirements between the SGC and the IGC 4
Overrepresentation of species-level bins 5
Distribution of the annotated 16S rRNA genes between predicted bins 6
Distribution of annotated elements acquired during genome evolution between predicted bins 8
SUPPLEMENTARY TABLES 10
Table S1. Description of the 47 strains included in the SGC. 10
Table S2. Taxonomy of the 47 strains included in the SGC. 11
Table S3. Theoretical abundance profiles of the 47 strains across the 40 samples. 12
Table S4. Summary of genes and bin assignments along the creation of the gold standard. 14
SUPPLEMENTARY FIGURES 15
Figure S1. Tree representation of the taxonomy of the strains included in the SGC 16
Figure S2. Community structure of the SGC at the genus-level and alpha-diversity at the species-level of the 40 simulated samples. 16
Figure S3. Input requirements for the benchmarked binners 17
REFERENCES 18

SUPPLEMENTARY METHODS

Selection criteria for taxonomy-independent binners

Abundance-based binners. This first category comprises MGS-CANOPY (1) and MSPMINER (2) which has the particularity to distinguish core, accessory and shared genes. We selected these two binners since they have been specifically developed to group genes and previously applied to a non-redundant gut microbiota gene catalog (extended version of MetaHIT for MGS-CANOPY and IGC for MSPMINER). Although their strategies are similar, we found it necessary to consider both methods since no independent benchmarking results are available. We did not include other abundance-based binners in this category since our main interest is to evaluate if incorporating new types of information (such as gene composition) or combining the results of different solutions could improve the binning of the IGC genes.

Hybrid binners. Several binners implementing a hybrid approach have been published in the last years. To make our selection, we used the following main criteria: (i) published and reviewed binner at the time of this study; (ii) ability to use multiple samples; (iii) sufficient scalability to group the SGC genes; (iv) independence of a manual initialization for the number of bins; (v) ability to take pre-computed abundance profiles as input; (vi) relatively easy installation with non-proprietary requirements. Therefore, six binners lie in this second category of approaches: SOLIDBIN (3), COCACOLA (4), CONCOCT (5), METABAT (6), MAXBIN (7) and MYCC (8). Except for the first one which was recently published, all these binners were benchmarked (9, 10) in the first CAMI challenge. MAXBIN and METABAT were ranked in the top 2 for at least one of the tested datasets, and along with CONCOCT, they were used for recent MAG reconstructions (11–13). Moreover, CONCOCT implements a strategy different from the other hybrid binners, being based on a variational Bayesian approach. From the methods tested on the CAMI challenge, we also included COCACOLA to evaluate its performance on our dataset (containing for example more samples than the datasets used in the first CAMI challenge) and MYCC since it has the particularity to propose and to have tested different kmer sizes (such as 5p6mer). Finally, to include a newly published hybrid approach, we selected SOLIDBIN which distinguishes itself from COCACOLA by its semi-supervised spectral clustering method, allowing also to add constraints based on Sequence Feature Similarity (SFS). As detailed in Table 1 and in our results, it should however be noted that we present the results of the naïve mode of SOLIDBIN since the computational requirements were already very high and are increased with the SFS mode.

Integrative binners. We only included DAS TOOL (14) in this third category. It selects bins from several bin sets by iteratively computing a scoring function based on Single-Copy Marker Genes (SCMG). This binner was also used in MAG reconstructions (12) and has been shown to recover the most high-quality bins on the CAMI high-complexity dataset from the output of MAXBIN, METABAT, MYCC, CONCOCT, METAWATT (15), and BINSANITY (16). These last two binners were not included in our analysis since they did not meet at least one of the above criteria. To our knowledge, other available integrative binners are either also based on SCMG or essentially focus on refining bins.

Computing environment and resources

This work was performed using the computing facilities of the CC LBBE/PRABI. All runs were realized on this computing cluster (Linux environment). We created CONDA environments to install binners with python packages dependencies and to ensure their usability independently of the used node (COCACOLA, CONCOCT, DAS TOOL, MYCC and SOLIDBIN). The time and memory requirements were measured using either the SACCT command accessible through the SLURM Workload Manager, or the verbose mode of the GNU TIME program available on Linux environments. The detailed memory peak corresponds to the Maximum Resident Set Size (MaxRSS) and the elapsed time is equivalent to the wall clock time.

For the benchmarking on the SGC, we set the number of threads to 16 when possible. Four binners (MYCC, SOLIDBIN, MSPMINER and COCACOLA) do not provide a configurable input parameter to set the number of threads. For those binners, we set the environment variables ensuring a maximum number of 16 usable threads and, when necessary, modified the hardcoded parameters of external tools called by the binner (*e.g.* FRAGGENESCAN (17) and HMMER (18) used for the bin number estimation by SOLIDBIN and COCACOLA). For MSPMINER, setting the environment variable is the same as having a configurable parameter since the code is parallelized with OPENMP, whereas for the others only part of the code is influenced by this parameter.

For the application to the IGC, we first launched MAXBIN (with a gene length filter set to 500 bp) and MGS-CANOPY (with the early stopping parameter set to 0) using 32 threads and a wall time of 7 days. We programmed an automatic interruption of the computation for MGS-CANOPY after 6 days and 20 hours. Unfortunately, we did not obtain binning results for MAXBIN after 7 days of computation. We then launched DAS TOOL, using default parameters, on the published binning results of MSPMINER and on the results obtained on the IGC with MGS-CANOPY.

Selection of 16S rRNA, plasmid and prophage genes to evaluate their assignment

Considering the genomes of the 47 selected strains, the full set of 16S rRNA genes contains 156 genes. The clustering step generated 35 clusters of 16 rRNA genes, including 9 composed of only one sequence (singleton clusters). Among the remaining 26 clusters, 15 clusters contained only 16 rRNA genes from one strain; 9 from two strains; and 2 from three strains. We kept only the representative sequence of each cluster in our simulated non-redundant gene catalog. As explained below in the construction process of our gold standard, we considered these representative sequences to be *shared* by all the strains associated to their cluster (*e.g.* 9 genes are shared between two strains). For each of the selected binners, we have compared the assignment of the 35 non-redundant 16S rRNA genes in the SGC with their expected distribution.

Given that plasmids are present and annotated in a subset of the strains included in our SGC, we studied 88 non-redundant genes of known plasmids annotated in the following strains: *Bifidobacterium longum* NCC2705, *B. longum* BG7, *Escherichia coli* O157:H7 str. Sakai, and *Buchnera aphidicola* str. APS. Regarding the prophage genes, taking into account that they are more common

and can have various annotations, we focused our analysis on 30 non-redundant prophage genes found in the genome of eight strains. However, it should be noted that it represents a small subset of the total number of prophage genes present in the SGC. We grouped these eight strains into two categories: (i) belonging to the same genus – 2 strains of *Methanobrevibacter* and of *Cellulomonas*; (ii) belonging to the same species – 2 strains of *B. longum* and 2 *of Fusobacterium nucleatum*. Moreover, except for 1 prophage gene shared among the strains of *B. longum*, all prophage and plasmid genes are specific to one strain present in the SGC.

SUPPLEMENTARY ANALYSES

Computational requirements on the SGC

Although our main interest was to evaluate binners based on their ability to accurately bin genes from a non-redundant catalog into species-level bins, we propose an overall comparison of the computational resource requirements of the benchmarked binners. Nevertheless, it should be noted that binners were run on a shared computer cluster, therefore the comparison results only aim to give a global idea on the required computational resources. For the hybrid binners launched using two gene length thresholds, both runs were realized on the same machine. Globally, we can distinguish binners by three main groups representing combinations of time (elapsed time) and memory requirements (memory peak) on the SGC. The two fastest are the abundance-based binners. MGS-CANOPY and MSPMINER output binning results in less than 2 minutes, using between less than 1 Gb (MGS-CANOPY) and up to 6.6 Gb (MSPMINER). The second group corresponds to the integrative and four hybrid binners (DAS TOOL, CONCOCT, MAXBIN, COCACOLA, METABAT) requiring between 2-29 min and less than 2 Gb to cluster the SGC genes. While runs with the two gene length thresholds are both included in this group, COCACOLA is the only one for which the elapsed time did not double (12 to 19 min) when passing from the 1000 bp to the 500 bp threshold. Indeed, the time varies from 2 to 4 min between both thresholds for CONCOCT and between 14 to 29 min for MAXBIN. The last group is composed of MYCC and SOLIDBIN, both requiring more than 2 h (SOLIDBIN_1000 and up to 20 h with MYCC_5P6_500) of computation, using between 9 Gb (MYCC_5P6_1000) and 482 Gb of memory (SOLIDBIN_500). For both methods, memory usage is multiplied by 3.5 (SOLIDBIN) up to 5 (MYCC), and the elapsed time is multiplied by 2.5 (MYCC) up to 6.5 (SOLIDBIN) between both thresholds.

Evolution of the computational requirements between the SGC and the IGC

In order to compare the time and memory requirements between both catalogs, we ran MGS-CANOPY on the IGC. Then, we launched DAS TOOL with the computed MGS-CANOPY results and MSPMINER published (2) results as inputs. Considering the size of the catalog, we used 32 threads for the computation on the IGC whereas we performed the benchmarking on the SGC with 16 threads. The relative computational requirement tendencies globally differ between the two gene catalogs. In terms of memory peak, MGS-CANOPY requires the least memory (<1 Gb) on the SGC whereas it used the most

on the IGC (201 Gb). However, on both catalogs, MSPMINER (6.6 Gb on the SGC and 74 Gb on the IGC) used more memory than DAS TOOL (1.5 Gb on the SGC and 40 Gb on the IGC). As for the elapsed time, MGS-CANOPY was the fastest on the SGC (<1 min) but the longest on the IGC. We interrupted it after 6 days and 20 hours to stop the clustering and start the merging of clusters. We obtained the partial binning results 1h40 afterwards. On both catalogs, MSPMINER was faster (1 min 23 sec on the SGC and 160 min on the IGC) than DAS TOOL (3 min 04 sec and 345 min).

Overrepresentation of species-level bins

Overrepresentation of species-level bins by the abundance-based and hybrid selected binners. All of the four species represented with two bins by MAXBIN are also split by MSPMINER in three to six bins. As shown in Figure 5B**,** three species (*Blautia obeum*, *F. nucleatum and Anaerobutyricum hallii*) out of these four are composed of genes belonging to two different strains (identified as sharing low to medium ANI and TETRA values for the first two species), and are associated to a relatively low average GC content (< 43%). For each of these species, both binners output at least one bin essentially composed of genes belonging specifically to each strain and of shared genes between the two strains. They also recover bins representing multiple species which share several genes (*e.g. B. obeum* A2-162 and *Blautia* sp. Marseille-P3087 in msp_07 and in maxbin.006 – Figure 4C-D). Nevertheless, as shown in Figure 4A and by the grey bar plot in Figure 5B, we indeed expect two to three bins to be representative of the species sharing more than 4% of genes (*B. obeum*, *Blautia* sp. Marseille-P3087, *Ruminococcus* sp. SR1/5, *Blautia* sp. KLE 1732 and *Dorea formicigenerans*). In other cases, MSPMINER tends to output two bins for a same strain (*e.g. A. hallii* EH1 with msp_37 and msp_35). Even though DAS TOOL recovers only one bin for each of the three species, they correspond to a partial bin (Figure 4B) selected from MSPMINER (*A. hallii*) or from MGS-CANOPY (*B. obeum and F. nucleatum*). These bins are mainly composed of genes specific to only one strain, along with shared genes between the two strains. The last species represented with two bins by MAXBIN is *Ruminococcus* sp. SR1/5 and is composed of genes from only one strain of this species. Despite this and similarly to our gold standard, MSPMINER overrepresents it with three bins (msp_29, msp_13 and msp_11) containing genes from *Blautia* sp. KLE 1732 which share a large proportion of genes and high ANI and TETRA values with *Ruminococcus* sp. SR1/5 (Figure 5B). Several genes from these two species are also mixed by DAS TOOL and MAXBIN in one of their representative bins for *Ruminococcus* sp. SR1/5.

Overrepresentation of species-level bins by the abundance-based selected binner only. We have identified three cases among the species that are only overrepresented by MSPMINER (Figure 5B): (i) three species (*Anaerotruncus colihominis*, *B. longum*, and *E. coli*) composed of genes belonging to two strains with a medium to high average GC content (47-63%), including one with high ANI and TETRA values between its strains (*B. longum)*; (ii) two species (*Subdoligranulum variabile* and *Faecalibacterium prausnitzii*) from the same family with a similar high average GC content (56-58%), sharing low ANI and TETRA values; (iii) six species (*D. formicigenerans, [Eubacterium] rectale, [Ruminococcus] gnavus, Methanobrevibacter oralis, Campylobacter jejuni and Blautia* sp. Marseille-

P3087*)* containing genes from a single strain of the species and including two species (*D. formicigenerans and Blautia* sp. Marseille-P3087) sharing a large proportion of genes with another species. In the first case, MSPMINER outputs bins constituted of a mixture of genes specific to both strains. It recovers at least a bin per strain (essentially genes from this strain and shared genes between strains), aside from *B. longum* for which none of the bins are mainly composed of genes specific to *B. longum* NCC2705. In contrast with the previous section, a single bin is recovered by MAXBIN and DAS TOOL (complete bin selected from MSPMINER) for these three species. These single bins are composed of genes specific to both strains and of a majority of shared genes between both strains. In the second case, while the two species are represented with distinct bins by MAXBIN (Figure 4C), MSPMINER outputs two bins composed of a mixture of genes specific to each species (Figure 4D). By selecting a complete bin from MGS-CANOPY (CAG023) and a partial one from MSPMINER (msp_15_sub), DAS TOOL recovers a separate bin for each species (Figure 4B).

In the third case, three out of the six species (*D. formicigenerans, E. rectale and R. gnavus*) belong to the family of Lachnospiraceae (Supplementary Figure S1) and are each overrepresented with two to three bins by MSPMINER. As shown in Figure 4D, each species is represented by one main bin (msp_26, msp_19 and msp_22), containing also genes from at least one of the other two species. As for MAXBIN, it outputs three bins (maxbin.008, maxbin.011 and maxbin.004) which are each mainly composed of genes belonging to one species, except for maxbin.011 representing *E. rectale* and containing almost 10% of the genes of *Ruminococcus* sp. SR1/5*.* Regarding the other three species, in the results of MSPMINER (Figure 4D) *M. oralis* (msp_34), an Archaea*,* has some of its genes in the representative bin of two strains belonging to the super-kingdom of Bacteria*: C. jejuni* (msp_49) and *F. nucleatum* (msp_45 and msp_39). MAXBIN outputs a single bin for *C. jejuni* (maxbin.031) *and* for *M. oralis* (maxbin.024*)*. Nonetheless, the one from *M. oralis* is also constituted of genes from *Methanobrevibacter smithii* (same genus) and from *Lactobacillus acidophilus* (Bacterium). Similarly, MSPMINER recovers four bins for *Blautia* sp. Marseille-P3087 (msp_20, msp_32, msp_07 and msp_21) while MAXBIN outputs a single bin for this strain (maxbin.006). Yet, these five bins are composed of genes specific to *B. obeum* (Figure 4). DAS TOOL selected one partial bin of MGS-CANOPY also containing genes from *B. obeum.* However, for the other five species of this category, DAS TOOL manages to recover separated bins (Figure 4B). It selected either one complete bin from MGS-CANOPY (CAG032 for *M. oralis* and CAG014 for *R. gnavus*), a partial one from MGS-CANOPY (CAG022_sub for *D. formicigenerans* and CAG037_sub for *C. jejuni*) or from MSPMINER (msp_19_sub for *E. rectale*).

Distribution of the annotated 16S rRNA genes between predicted bins

The one gene unassigned by MAXBIN belongs to *Enterocloster lavalensis* and is filtered out due to a gene length of less than 500 bp, whereas the one unassigned by MSPMINER belongs to *Ruminococcus* sp. SR1/5 and is kept after filtering too rare genes but removed afterwards. While the former gene is correctly assigned by MSPMINER and DAS TOOL, the latter is correctly assigned by MAXBIN but not by DAS TOOL. Indeed, this gene was also removed from the results of MGS-CANOPY after we selected bins with

more than 700 genes. Besides, we do not present the output of DAS TOOL based on the binning results of hybrid binners since in that case, the results of DAS TOOL were identical to those showed in Figure 4.

Globally, among their assigned 16S rRNA genes, MSPMINER correctly assigns 85%, whilst DAS TOOL and MAXBIN correctly assign 77% and 59%. This proportion remains similar when considering the whole set of non-redundant 16S rRNA genes, except for DAS TOOL for which it goes down to 69%. MAXBIN incorrectly assigns ten genes by placing them in a bin representative of a species of the same genus (*e.g.* 16S rRNA gene of *Bacteroides fragilis* in the representative bin of *Bacteroides ovatus*); family (*e.g.* 16S rRNA gene of *Coprococcus eutactus* in the representative bin of *D. formicigenerans*); order (*e.g.* 16S rRNA gene of *B. aphidicola* in the representative bin of *E. coli*); or super-kingdom (*e.g.* 16S rRNA gene of *E. rectale* in the representative bin of *Bacteroides vulgatus*). As for DAS TOOL, it incorrectly assigns only one gene (from *B. aphidicola*) by associating it, similarly to MAXBIN, to one of the representative bins of *E. coli* (msp_04). Even though this bin has been selected from the results of MSPMINER, the latter gene is considered as correctly assigned by MSPMINER given that this binner manages to also assign it to the representative bin of *B. aphidicola.*

Consequently, out of the 34 genes assigned by MSPMINER, none is incorrectly assigned, 29 are correctly assigned and five are partially correctly assigned. Each of these partially correctly assigned genes are shared between two or three strains from the same genus (*e.g. Cellulomonas timonensis* and *Cellulomonas massiliensis*) or order (*e.g. Roseburia intestinalis*, *Butyrivibrio crossotus*, and *A. colihominis)*. Among the correctly assigned genes, four are also shared between two strains from the same species (*B. longum*, *A. colihominis*, *F. nucleatum*, and *A. hallii*); one gene between two strains from the same genus (*M. smithii and M. oralis*); and one gene between two strains from the same family (*Enterobacter cloacae* and *E. coli* belonging to the family of *Enterobacteriaceae*). The 16S rRNA genes correctly assigned by MSPMINER are placed in at least one of the representative bins of each expected species. Some 16S rRNA genes are even correctly placed in all the representative bins of their expected species. However, this can sometimes lead to a partially incorrect assignment if some of those bins are also representative of another species. For instance, the 16S rRNA gene of *F. prausnitzii* is correctly assigned to the two bins representative of this species (msp_15 and msp_16, Figure 4D), including one containing mostly genes from *S. variabile*. In that case, DAS TOOL assigns this latter gene only to the main representative predicted bin of *F. prausnitzii* (CAG023, Figure 4B). This is essentially explained by the fact that DAS TOOL outputs non-redundant bins, and therefore assigns each gene to a single bin. All of the genes partially correctly assigned by MSPMINER are either also partially correctly assigned by DAS TOOL or unassigned (16S rRNA gene shared between *B. obeum*, *Blautia* sp. Marseille-P3087 and *Blautia* sp. KLE 1732). Moreover, three of the 16S rRNA genes correctly assigned by MSPMINER are also either unassigned (*F. nucleatum*) or partially correctly assigned by DAS TOOL (one gene shared between strains belonging to the family *Enterobacteriaceae* and one gene shared between strains belonging to the genus *Methanobrevibacter*). Similar observations can be made for MAXBIN which also outputs single assignments. The 16S rRNA genes partially correctly assigned by MSPMINER are either incorrectly (16S rRNA gene of *Anaerostipes caccae* and of *Eubacterium ventriosum*) or partially correctly assigned by MAXBIN. Among the 20 correctly assigned genes by MAXBIN, the 16S rRNA gene shared between both strains of the genus *Methanobrevibacter* is assigned to the bin maxbin.024,

corresponding to the main representative bin of *M. oralis*, *M. smithii* and *L. acidophilus* (Figure 4C). Indeed, even though they belong to different super-kingdoms (two Archaea and one Bacterium), more than 66% of the genes of each of these three species are included in this bin.

Distribution of annotated elements acquired during genome evolution between predicted bins

Among the selected and assigned plasmid and prophage genes, all three binners output correct assignments but we still noted some interesting cases. For instance, MSPMINER recovers three representative bins for *E. coli* and for *B. longum* (Figure 5B) and places the corresponding plasmid genes in some of or in all the respective representative bins. In the former case, the 75 plasmid genes belonging to *E. coli* are assigned to the msp_08, of which 28 are also assigned to the msp_04. Besides, this latter bin was the one selected by DAS TOOL to be representative of *E. coli*, explaining the loss of 47 plasmid genes in the results of DAS TOOL.

As for prophage genes, while MAXBIN places in the same bin (maxbin.013) the prophage genes from the two strains belonging to species *B. longum,* it also does the same for the genus *Methanobrevibacter*. In fact, even though they belong to different species, both strains of this genus are included in a single bin (maxbin.024, Figure 4C). Besides, only genes from the strain belonging to *C. timonensis* are assigned by MAXBIN (maxbin.037) since prophage genes from *C. massiliensis* do not pass the gene length threshold. In contrast with the case of *B. longum*, prophage genes from *F. nucleatum* are split by MAXBIN into the two main representative bins of this species. Those genes are assigned to the bin including the most genes specific to each strain (maxbin.035 for *F. nucleatum* subsp. animalis ATCC 51191 and maxbin.036 for *F. nucleatum* subsp. fusiforme ATCC 51190). Likewise, as shown in Figure 4D and Figure 5B, MSPMINER also splits the genes belonging to *F. nucleatum* into four representative bins (msp_45, msp_39, msp_28 and msp_34), containing also genes belonging to *M. oralis*. Out of these four, the main bin (msp_28) is composed of a majority of genes of *F. nucleatum* subsp. animalis ATCC 51191 and contains the prophage genes from this strain. The remaining prophage genes are repeatedly placed into the four representative bins. Similar observations can be made for the genes belonging to *Methanobrevibacter*. Prophage genes from *M. smithii* are placed in the representative bin of the species (msp_50) and the single gene of *M. oralis* is repeated in three bins shared with *F. nucleatum* (msp_45, msp_39 and msp_34). Interestingly, DAS TOOL does not mix the prophage genes from these genera and places them into the representative bin of each species. As illustrated in Figure 4B for *Methanobrevibacter*, DAS TOOL selected the representative bin of *M. smithii* from the results of MSPMINER (msp_50) and one bin predicted by MGS-CANOPY (CAG032) containing only genes belonging to *M. oralis.* Similarly, a partial bin was selected from the results of MGS-CANOPY (CAG024_sub) and is composed of a majority of genes belonging to the strain *F. nucleatum* subsp. animalis ATCC 51191. Therefore, only prophage genes from this latter strain were assigned by DAS TOOL. Finally, DAS TOOL and MSPMINER correctly assigned the genes from both strains of *B. longum* to the same bins. However, genes belonging to *B. longum* were repeatedly placed in two to three bins by MSPMINER (msp_43, msp_47 and msp_46), and only in one (msp_47) by DAS TOOL. Both binners also

correctly assigned the prophage genes of the strains belonging to *Cellulomonas* to separate bins representing each species (CAG011 and CAG015 for DAS TOOL, msp_18 and msp_27 for MSPMINER).

SUPPLEMENTARY TABLES

Table S1. Description of the 47 strains included in the SGC. The main selection criteria for the 47 strains are described: average GC content, genome completeness, known plasmid or prophage region, number of genes in the reference genome (indicative values computed in 2018), closely related genomes (ANI > 95% and TETRA > 0.99), taxonomy, gene or genome size etc. The average GC content was computed for all chromosomes and plasmids. 26 strains are part of the core microbiota.

Strain	GC%	Genome compl.	Plasmids	Total #genes (+ plasmid)	Other selection criteria
***Alistipes putredinis* DSM 17216**	53.3	Contigs	-	2426	-
***Anaerobutyricum hallii* DSM 3353**	38.2	Contigs	-	3300	Species with two strains
***Anaerobutyricum hallii* EH1**	38.6	Complete	-	3198	
***Anaerostipes caccae* DSM 14662**	44.3	Contigs	-	3546	-
***Anaerotruncus colihominis* 2789STDY5834939**	54.1	Contigs	-	3782	Species with two strains
***Anaerotruncus colihominis* DSM 17241**	54.2	Contigs	-	3777	
***Bacteroides fragilis* YCH46**	38.4	Complete	pBFY46	4670 + 47	-
***Bacteroides ovatus* ATCC 8483**	41.9	Complete	-	4996	-
***Bacteroides vulgatus* ATCC 8482**	42.2	Complete	-	4333	-
***Bifidobacterium longum* BG7**	63.2	Complete	pRY68	2126 + 2	Species with two closely related strains (ANI=98.12 and TETRA=0.998), prophage region in BG7
***Bifidobacterium longum* NCC2705**	62.5	Complete	pBLO1	1797 + 2	
***Blautia hansenii* DSM 20583**	39	Contigs	-	3070	-
***Blautia obeum* A2-162**	42.6	Contigs	-	3210	Species with two not closely related strains (ANI=82.93 and TETRA=0.975)
***Blautia obeum* ATCC 29174**	41.6	Contigs	-	3502	
***Blautia* sp. KLE 1732**	44.2	Contigs	-	3395	Closely related with *Ruminococcus* sp. SR1/5 (ANI=97.56, TETRA=0.999)
***Blautia* sp. Marseille-P3087**	42.5	Contigs	-	3153	Long genes
***Buchnera aphidicola* str. APS**	27.9	Complete	pLeu + pTrp	606 + 7 + 4	Small genome
***Butyrivibrio crossotus* DSM 2876**	37.7	Contigs	-	2488	-
***Campylobacter jejuni* ATCC 700819**	30.5	Complete	-	1668	Small genome
***Candida albicans* SC5314**	33.5	Complete	-	6263	Fungus
***Cellulomonas massiliensis* JC225**	74.8	Contigs	-	3175	High GC%
***Cellulomonas timonensis* SN7**	72.4	Contigs	-	3710	
***[Clostridium] leptum* DSM 753**	50.2	Contigs	-	3081	-
***Coprococcus eutactus* ATCC 27759**	43.1	Contigs	-	2728	-
***Dorea formicigenerans* ATCC 27755**	41	Contigs	-	3348	-
***Enterobacter cloacae* FDA-CDC-AR_0164**	54.4	Complete	-	4771	Short genes
***Enterocloster lavalensis* NLAE-zl-G277**	55.3	Contigs	-	5782	Long genes
***Escherichia coli* K12 substr. MG1655**	50.8	Complete	-	4498	Species with 2 strains, genes from this species have been classified into three classes of codon usage (19, 20)
***Escherichia coli* O157:H7 str. Sakai**	47.2	Complete	pO157 + pOSAK1	5358 + 85 + 3	
***[Eubacterium] rectale* ATCC 33656**	41.5	Complete	-	3366	-
***Eubacterium ventriosum* ATCC 27560**	34.9	Contigs	-	2618	-
***Ezakiella peruensis* M6.X2**	36.9	Contigs	-	1608	Small genome
***Faecalibacterium prausnitzii* A2-165**	56.4	Contigs	-	3075	Same family as *Subdoligranulum variabile* and not closely related (ANI=72.67 and TETRA=0.908)
***Fusobacterium nucleatum* subsp. animalis ATCC 51191**	27.5	Contigs	-	2827	Species with 2 not closely related strains (ANI=91.51 and TETRA=0.996)
***Fusobacterium nucleatum* subsp. fusiforme ATCC 51190**	27.2	Contigs	-	1873	
***Helicobacter pylori* 26695**	38.9	Complete	-	1555	Small genome
***Lactobacillus acidophilus* LA1**	34.7	Complete	-	2002	Short genes
***Methanobrevibacter oralis* JMR01**	27.8	Contigs	-	2027	Archaea, Low GC%
***Methanobrevibacter smithii* ATCC 35061**	31	Complete	-	1793	
***Parabacteroides distasonis* ATCC 8503**	45.1	Complete	-<br>-	4081	-
***Roseburia intestinalis* L1-82**	42.6	Contigs	-	4347	-
Rotavirus A	34.7	Complete		12	Virus
***Ruminococcus bromii* YE282**	39.1	Contigs	-	2569	-
***[Ruminococcus] gnavus* AGR2154**	42.5	Contigs	-	3714	-
***Ruminococcus* sp. SR1/5**	44.2	Contigs	-	3313	Closely related with *Blautia* sp. KLE 1732
***Subdoligranulum variabile* DSM 15176**	57.9	Contigs	-	3114	Same family as *Faecalibacterium prausnitzii* A2-165 and not closely related
***Tyzzerella nexilis* DSM 1787**	40.1	Contigs	-	3952	-

Table S2. Taxonomy of the 47 strains included in the SGC.

Accession	Super-kingdom	Phylum	Class	Order	Family	Genus	Species	Strain
**GCF_000154465.1**	Bacteria	Bacteroidetes	Bacteroidia	Bacteroidales	Rikenellaceae	Alistipes	Alistipes putredinis	DSM 17216
**GCF_000154305.1**	Bacteria	Firmicutes	Clostridia	Clostridiales	Lachnospiraceae	Anaerostipes	Anaerostipes caccae	DSM 14662
**GCF_001404495.1**	Bacteria	Firmicutes	Clostridia	Clostridiales	Ruminococcaceae	Anaerotruncus	Anaerotruncus colihominis	2789STDY5834939
**GCF_000154565.1**	Bacteria	Firmicutes	Clostridia	Clostridiales	Ruminococcaceae	Anaerotruncus	Anaerotruncus colihominis	DSM 17241
**GCF_000009925.1**	Bacteria	Bacteroidetes	Bacteroidia	Bacteroidales	Bacteroidaceae	Bacteroides	Bacteroides fragilis	YCH46
**GCF_001314995.1**	Bacteria	Bacteroidetes	Bacteroidia	Bacteroidales	Bacteroidaceae	Bacteroides	Bacteroides ovatus	ATCC 8483
**GCF_000012825.1**	Bacteria	Bacteroidetes	Bacteroidia	Bacteroidales	Bacteroidaceae	Bacteroides	Bacteroides vulgatus	ATCC 8482
**GCF_001293145.1**	Bacteria	Actinobacteria	Actinobacteria	Bifidobacteriales	Bifidobacteriaceae	Bifidobacterium	Bifidobacterium longum	BG7
**GCF_000007525.1**	Bacteria	Actinobacteria	Actinobacteria	Bifidobacteriales	Bifidobacteriaceae	Bifidobacterium	Bifidobacterium longum	NCC2705
**GCF_002222595.2**	Bacteria	Firmicutes	Clostridia	Clostridiales	Lachnospiraceae	Blautia	Blautia hansenii	DSM 20583
**GCA_000210015.1**	Bacteria	Firmicutes	Clostridia	Clostridiales	Lachnospiraceae	Blautia	Blautia obeum	A2-162
**GCF_000153905.1**	Bacteria	Firmicutes	Clostridia	Clostridiales	Lachnospiraceae	Blautia	Blautia obeum	ATCC 29174
**GCF_000466565.1**	Bacteria	Firmicutes	Clostridia	Clostridiales	Lachnospiraceae	Blautia	Blautia sp. KLE 1732	KLE 1732
**GCF_900120195.1**	Bacteria	Firmicutes	Clostridia	Clostridiales	Lachnospiraceae	Blautia	Blautia sp. Marseille-P3087	Marseille-P3087
**GCF_000009605.1**	Bacteria	Proteobacteria	Gammaproteobacteria	Enterobacterales	Erwiniaceae	Buchnera	Buchnera aphidicola	str. APS
**GCF_000156015.1**	Bacteria	Firmicutes	Clostridia	Clostridiales	Lachnospiraceae	Butyrivibrio	Butyrivibrio crossotus	DSM 2876
**GCF_000009085.1**	Bacteria	Proteobacteria	Epsilonproteobacteria	Campylobacterales	Campylobacteraceae	Campylobacter	Campylobacter jejuni	ATCC 700819
**GCF_000182965.3**	Eukaryota	Ascomycota	Saccharomycetes	Saccharomycetales	Debaryomycetaceae	Candida	Candida albicans	SC5314
**GCF_000312005.1**	Bacteria	Actinobacteria	Actinobacteria	Micrococcales	Cellulomonadaceae	Cellulomonas	Cellulomonas massiliensis	JC225
**GCF_900046455.1**	Bacteria	Actinobacteria	Actinobacteria	Micrococcales	Cellulomonadaceae	Cellulomonas	Cellulomonas timonensis	SN7
**GCF_900102595.1**	Bacteria	Firmicutes	Clostridia	Clostridiales	Lachnospiraceae	Enterocloster	Enterocloster lavalensis	NLAE-zl-G277
**GCF_002556665.1**	Bacteria	Firmicutes	Clostridia	Clostridiales	Ruminococcaceae		[Clostridium] leptum	DSM 753
**GCF_000154425.1**	Bacteria	Firmicutes	Clostridia	Clostridiales	Lachnospiraceae	Coprococcus	Coprococcus eutactus	ATCC 27759
**GCF_000169235.1**	Bacteria	Firmicutes	Clostridia	Clostridiales	Lachnospiraceae	Dorea	Dorea formicigenerans	ATCC 27755
**GCF_003071645.1**	Bacteria	Proteobacteria	Gammaproteobacteria	Enterobacterales	Enterobacteriaceae	Enterobacter	Enterobacter cloacae	FDA-CDC-AR_0164
**GCF_000005845.2**	Bacteria	Proteobacteria	Gammaproteobacteria	Enterobacterales	Enterobacteriaceae	Escherichia	Escherichia coli	K12 substr. MG1655
**GCF_000008865.1**	Bacteria	Proteobacteria	Gammaproteobacteria	Enterobacterales	Enterobacteriaceae	Escherichia	Escherichia coli	O157:H7 str. Sakai
**GCF_000173975.1**	Bacteria	Firmicutes	Clostridia	Clostridiales	Lachnospiraceae	Anaerobutyricum	Anaerobutyricum hallii	DSM 3353
**GCF_900209925.1**	Bacteria	Firmicutes	Clostridia	Clostridiales	Lachnospiraceae	Anaerobutyricum	Anaerobutyricum hallii	EH1
**GCF_000020605.1**	Bacteria	Firmicutes	Clostridia	Clostridiales	Lachnospiraceae		[Eubacterium] rectale	ATCC 33656
**GCF_000153885.1**	Bacteria	Firmicutes	Clostridia	Clostridiales	Eubacteriaceae	Eubacterium	Eubacterium ventriosum	ATCC 27560
**GCF_900215725.1**	Bacteria	Firmicutes	Tissierellia			Ezakiella	Ezakiella peruensis	M6.X2
**GCF_000162015.1**	Bacteria	Firmicutes	Clostridia	Clostridiales	Ruminococcaceae	Faecalibacterium	Faecalibacterium prausnitzii	A2-165
**GCA_000220825.1**	Bacteria	Fusobacteria	Fusobacteriia	Fusobacteriales	Fusobacteriaceae	Fusobacterium	Fusobacterium nucleatum	subsp. animalis ATCC 51191
**GCF_000279975.1**	Bacteria	Fusobacteria	Fusobacteriia	Fusobacteriales	Fusobacteriaceae	Fusobacterium	Fusobacterium nucleatum	subsp. fusiforme ATCC 51190
**GCF_000008525.1**	Bacteria	Proteobacteria	Epsilonproteobacteria	Campylobacterales	Helicobacteraceae	Helicobacter	Helicobacter pylori	26695
**GCF_002286215.1**	Bacteria	Firmicutes	Bacilli	Lactobacillales	Lactobacillaceae	Lactobacillus	Lactobacillus acidophilus	LA1
**GCF_000529525.1**	Archaea	Euryarchaeota	Methanobacteria	Methanobacteriales	Methanobacteriaceae	Methanobrevibacter	Methanobrevibacter oralis	JMR01
**GCF_000016525.1**	Archaea	Euryarchaeota	Methanobacteria	Methanobacteriales	Methanobacteriaceae	Methanobrevibacter	Methanobrevibacter smithii	ATCC 35061
**GCF_000012845.1**	Bacteria	Bacteroidetes	Bacteroidia	Bacteroidales	Tannerellaceae	Parabacteroides	Parabacteroides distasonis	ATCC 8503
**GCF_000156535.1**	Bacteria	Firmicutes	Clostridia	Clostridiales	Lachnospiraceae	Roseburia	Roseburia intestinalis	L1-82
**GCF_000880735.1**	Viruses	Duplornaviricota	Resentoviricetes	Reovirales	Reoviridae	Rotavirus	Rotavirus A	Rotavirus A
**GCF_900101355.1**	Bacteria	Firmicutes	Clostridia	Clostridiales	Ruminococcaceae	Ruminococcus	Ruminococcus bromii	YE282
**GCF_000526735.1**	Bacteria	Firmicutes	Clostridia	Clostridiales	Lachnospiraceae	Mediterraneibacter	[Ruminococcus] gnavus	AGR2154
**GCA_000209835.1**	Bacteria	Firmicutes	Clostridia	Clostridiales	Ruminococcaceae	Ruminococcus	Ruminococcus sp. SR1/5	SR1/5
**GCF_000157955.1**	Bacteria	Firmicutes	Clostridia	Clostridiales	Ruminococcaceae	Subdoligranulum	Subdoligranulum variabile	DSM 15176
**GCF_000156035.2**	Bacteria	Firmicutes	Clostridia	Clostridiales	Lachnospiraceae	Tyzzerella	Tyzzerella nexilis	DSM 1787

Table S3. Theoretical abundance profiles of the 47 strains across the 40 samples.

Strain/Sample	1	2	3	4	5	6	7	8	9	10	11	12	13	14	15	16	17	18	19	20
A. putredinis **DSM 17216**	0.70	10.00	2.00	0.15	0.20	1.00	10.00	0.00	2.13	0.00	0.00	0.00	0.00	0.00	4.00	0.00	0.15	3.00	0.08	10.00
A. hallii **DSM 3353**	0.50	0.30	0.20	1.00	0.40	0.60	1.00	0.00	2.13	0.00	0.00	10.00	0.00	0.00	0.30	4.00	1.00	0.02	0.65	1.00
A. hallii **EH1**	0.40	0.30	0.20	1.00	0.60	0.40	1.00	0.00	2.13	0.00	0.00	0.00	0.00	0.40	0.01	0.20	0.01	0.00	0.40	0.03
A. caccae **DSM 14662**	0.90	10.00	1.00	0.20	0.70	0.30	1.00	0.00	2.13	0.00	0.00	10.00	0.00	0.90	10.00	16.00	0.02	3.00	0.30	0.01
A. colihominis **2789STDY5834939**	10.00	7.00	3.00	0.15	1.00	0.20	0.25	0.00	2.13	10.00	0.00	0.00	0.00	14.00	0.07	3.00	0.00	1.00	0.01	0.25
A. colihominis **DSM 17241**	0.70	7.00	3.00	0.10	0.90	0.20	0.25	0.00	2.13	10.00	0.00	0.00	0.00	0.70	7.00	3.00	22.00	0.09	0.20	0.00
B. fragilis **YCH46**	0.10	5.00	0.70	0.30	5.00	0.10	4.00	0.00	2.13	0.00	0.00	0.00	0.00	0.01	5.00	0.00	0.01	5.00	8.00	9.00
B. ovatus **ATCC 8483**	5.00	5.00	0.40	0.60	10.00	0.10	3.00	0.00	2.13	0.00	0.00	10.00	0.00	0.00	0.00	0.04	0.01	0.00	0.00	0.10
B. vulgatus **ATCC 8482**	1.00	4.00	0.50	0.50	5.00	0.10	3.00	0.00	2.13	0.00	0.00	0.00	0.00	1.00	4.00	0.50	0.00	0.09	9.00	3.00
B. longum **BG7**	1.00	3.00	7.00	0.10	0.15	2.00	7.00	8.33	2.13	10.00	0.00	0.00	0.00	1.00	0.00	7.00	0.20	0.25	13.00	0.00
B. longum **NCC2705**	0.20	3.00	10.00	0.10	0.10	3.00	7.00	8.33	2.13	10.00	0.00	0.00	0.00	0.20	3.00	0.01	0.00	0.10	3.00	0.00
B. hansenii **DSM 20583**	0.60	2.00	0.20	1.00	0.20	1.00	0.90	0.00	2.13	0.00	0.00	0.00	10.00	0.06	2.00	0.02	0.01	2.00	0.00	0.00
B. obeum **A2-162**	7.00	2.00	0.60	0.40	1.00	0.20	0.90	8.33	2.13	0.00	0.00	10.00	10.00	8.00	0.02	0.60	0.40	0.20	0.80	0.00
B. obeum **ATCC 29174**	2.00	1.00	0.40	0.60	0.90	0.25	0.70	8.33	2.13	0.00	0.00	0.00	10.00	3.00	1.00	0.00	2.00	0.00	0.00	0.70
Blautia **sp. KLE 1732**	0.25	1.00	0.90	0.25	0.70	0.30	0.70	8.33	2.13	0.00	0.00	0.00	10.00	0.00	12.00	0.90	0.25	0.70	0.00	0.07
Blautia **sp. Marseille-P3087**	0.20	1.00	0.50	0.50	0.50	0.50	0.60	0.00	2.13	0.00	0.00	0.00	10.00	0.20	0.00	0.50	0.50	0.50	0.50	0.06
B. aphidicola **str. APS**	0.10	1.00	0.10	28.00	0.10	28.00	0.10	0.00	2.13	0.00	10.00	0.00	0.00	0.01	1.00	0.10	0.00	0.01	28.00	0.10
B. crossotus **DSM 2876**	0.50	0.90	0.20	2.00	0.20	2.00	0.50	0.00	2.13	0.00	0.00	10.00	10.00	0.05	0.09	0.20	2.00	0.00	0.02	0.50
C. jejuni **ATCC 700819**	0.15	0.90	0.10	7.00	0.10	10.00	0.10	0.00	2.13	0.00	10.00	0.00	0.00	0.02	0.90	0.00	0.10	0.01	0.03	0.01
C. albicans **SC5314**	0.10	0.70	0.10	5.00	10.00	0.10	0.10	0.00	2.13	0.00	10.00	0.00	0.00	0.10	0.00	0.01	0.00	10.00	0.10	2.00
C. massiliensis **JC225**	0.30	0.70	28.00	0.10	0.50	0.50	5.00	0.00	2.13	10.00	0.00	0.00	0.00	0.30	0.70	0.00	0.01	3.00	0.00	0.00
C. timonensis **SN7**	0.10	0.60	10.00	0.10	2.00	0.20	5.00	0.00	2.13	10.00	0.00	0.00	0.00	0.00	0.00	0.00	10.00	0.10	0.20	0.00
C. leptum **DSM 753**	0.50	0.60	1.00	0.20	0.40	0.60	0.20	0.00	2.13	0.00	0.00	10.00	0.00	0.00	0.60	40.00	0.20	0.00	0.60	0.30
C. eutactus **ATCC 27759**	3.00	0.50	0.70	0.30	0.25	0.90	0.50	0.00	2.13	0.00	0.00	0.00	10.00	1.00	0.01	0.07	0.70	0.06	0.09	0.05
D. formicigenerans **ATCC 27755**	3.00	0.50	0.30	0.70	0.30	0.70	0.50	0.00	2.13	0.00	0.00	10.00	10.00	5.00	33.00	0.50	0.00	0.30	0.09	0.60
E. cloacae **FDA-CDC-AR_0164**	0.40	0.50	4.00	0.10	4.00	0.10	0.10	0.00	2.13	10.00	0.00	0.00	0.00	0.40	0.00	0.00	26.00	0.10	0.10	27.00
E. lavalensis **NLAE-zl-G277**	0.15	28.00	5.00	0.10	7.00	0.10	0.40	0.00	2.13	10.00	0.00	0.00	10.00	0.02	2.59	0.05	3.00	0.00	0.60	0.40
E. coli **K12 substr. MG1655**	0.25	0.40	2.00	0.20	3.00	0.15	0.10	0.00	2.13	0.00	0.00	0.00	0.00	0.37	0.40	0.20	0.80	0.01	0.00	0.10
E. coli **O157:H7 str. Sakai**	0.10	0.40	1.00	0.20	7.00	0.10	0.10	0.00	2.13	0.00	0.00	0.00	0.00	0.10	0.40	0.10	0.01	0.09	0.00	0.01
E. rectale **ATCC 33656**	2.00	0.25	0.30	0.70	0.50	0.50	0.30	8.33	2.13	0.00	0.00	0.00	0.00	1.00	0.25	0.30	0.70	0.05	0.70	0.30
E. ventriosum **ATCC 27560**	4.00	0.25	0.15	3.00	0.20	1.00	1.00	8.33	2.13	0.00	10.00	0.00	0.00	4.00	0.00	0.30	0.00	0.30	6.00	0.09
E. peruensis **M6.X2**	0.20	0.20	0.15	2.00	0.10	10.00	0.15	0.00	2.13	0.00	0.00	0.00	0.00	0.02	0.08	0.00	6.00	0.10	0.00	0.15
F. prausnitzii **A2-165**	7.00	0.20	5.00	0.10	0.25	0.90	0.20	8.33	2.13	10.00	0.00	0.00	0.00	7.00	4.00	5.00	0.10	0.25	0.90	0.20
F. nucleatum **subsp. animalis ATCC 51191**	0.10	0.20	0.10	10.00	0.15	3.00	0.15	8.33	2.13	0.00	10.00	0.00	0.00	0.10	0.02	0.01	3.00	0.00	3.00	0.15
F. nucleatum **subsp. fusiforme ATCC 51190**	0.10	0.20	0.10	10.00	0.10	7.00	0.10	8.33	2.13	0.00	10.00	0.00	0.00	0.01	0.20	0.10	0.00	0.15	0.01	0.10
H. pylori **26695**	28.00	0.20	0.20	1.00	0.10	7.00	0.10	0.00	2.13	0.00	0.00	10.00	0.00	35.00	0.20	0.02	6.00	0.02	0.00	4.00
L. acidophilus **LA1**	0.20	0.15	0.10	3.00	0.10	5.00	2.00	0.00	2.13	0.00	10.00	0.00	0.00	0.00	0.00	0.00	5.00	0.10	2.00	0.00
M. oralis **JMR01**	0.30	0.15	0.10	7.00	0.10	4.00	28.00	0.00	2.13	0.00	10.00	0.00	0.00	0.03	0.15	0.10	0.02	17.00	0.09	0.00
M. smithii **ATCC 35061**	1.00	0.10	0.10	5.00	0.10	5.00	10.00	0.00	2.13	0.00	10.00	0.00	0.00	0.00	0.10	0.00	0.00	0.00	14.00	0.00
P. distasonis **ATCC 8503**	1.00	0.10	1.00	0.20	3.00	0.10	2.00	0.00	2.13	0.00	0.00	0.00	0.00	1.00	0.10	1.00	0.20	0.80	0.10	0.08
R. intestinalis **L1-82**	0.20	0.10	0.60	0.40	2.00	0.15	0.40	0.00	2.13	0.00	0.00	0.00	0.00	0.00	6.00	0.60	0.10	2.00	0.20	0.40
Rotavirus A	10.00	0.10	0.10	4.00	28.00	0.10	0.10	0.00	2.13	0.00	10.00	0.00	0.00	10.00	0.00	8.00	4.00	47.00	0.10	0.10
R. bromii **YE282**	0.10	0.10	0.25	0.90	0.20	1.00	0.20	0.00	2.13	0.00	0.00	0.00	0.00	0.10	0.00	0.25	0.00	0.00	6.00	38.00
R. gnavus **AGR2154**	5.00	0.10	0.50	0.50	1.00	0.25	0.60	0.00	2.13	0.00	0.00	10.00	10.00	4.00	0.80	0.07	5.00	1.00	0.00	0.60
Ruminococcus **sp. SR1/5**	0.10	0.10	0.90	0.25	0.60	0.40	0.20	8.33	2.13	0.00	0.00	10.00	0.00	0.01	0.01	0.00	0.50	0.60	0.04	0.04
S. variabile **DSM 15176**	0.90	0.10	7.00	0.10	0.30	0.70	0.20	8.33	2.13	10.00	0.00	0.00	0.00	0.90	0.00	7.00	0.01	0.00	0.90	0.20
T. nexilis **DSM 1787**	0.60	0.10	0.25	0.90	1.00	0.20	0.30	0.00	2.13	0.00	0.00	0.00	0.00	0.00	0.00	0.25	0.00	1.00	0.20	0.30

Strain/Sample	21	22	23	24	25	26	27	28	29	30	31	32	33	34	35	36	37	38	39	40
A. putredinis DSM 17216	0.00	0.00	0.01	0.20	0.00	0.00	0.00	0.00	0.10	0.10	0.00	2.00	0.00	0.00	0.00	0.00	0.00	0.00	0.00	0.00
A. hallii DSM 3353	0.80	0.50	0.50	0.00	0.10	20.00	0.00	0.00	0.09	0.20	0.00	0.00	7.00	0.00	1.00	0.00	0.00	0.00	0.00	0.00
A. hallii EH1	7.00	0.50	0.20	0.00	0.10	10.00	0.00	0.00	0.80	0.10	0.00	1.00	0.00	7.00	0.00	1.00	0.10	12.00	13.00	6.00
A. caccae DSM 14662	0.14	0.00	0.60	0.00	0.00	0.50	0.25	0.00	0.10	0.20	0.00	0.00	0.00	0.00	0.00	0.00	0.00	0.00	0.00	0.00
A. colihominis 2789STDY5834939	0.00	0.00	0.70	0.40	0.00	0.10	0.00	8.00	0.10	0.70	0.00	0.00	30.00	0.00	10.00	0.00	0.10	0.10	0.00	0.20
A. colihominis DSM 17241	0.30	0.00	0.70	0.50	0.00	0.10	0.00	6.00	0.00	0.01	0.00	0.50	0.00	30.00	0.00	10.00	0.00	0.00	0.00	0.00
B. fragilis YCH46	0.10	0.00	0.99	0.00	0.00	0.00	0.00	0.00	0.10	0.50	10.00	18.00	0.00	0.00	0.00	0.00	0.00	1.00	3.00	0.20
B. ovatus ATCC 8483	0.00	0.30	0.85	0.00	0.00	0.00	0.00	0.00	0.01	0.10	0.00	0.00	2.00	0.00	1.00	0.00	10.00	23.00	36.00	13.00
B. vulgatus ATCC 8482	45.00	0.00	0.80	0.00	0.00	0.00	0.00	0.00	0.50	0.00	0.00	0.00	0.00	2.00	0.00	1.00	27.00	10.00	3.00	20.00
B. longum BG7	0.30	0.01	0.95	7.00	0.00	0.00	0.00	0.00	0.10	20.00	30.00	20.00	1.00	0.00	40.00	0.00	0.10	0.30	0.20	0.20
B. longum NCC2705	0.60	0.08	0.95	10.00	0.00	0.00	0.00	0.00	0.00	20.00	15.00	10.00	0.00	1.00	0.00	40.00	4.00	0.00	0.10	3.00
B. hansenii DSM 20583	0.09	0.08	0.10	0.00	0.00	7.00	20.00	0.00	0.60	0.50	0.00	2.00	0.00	0.00	0.00	0.00	0.00	2.00	3.00	0.00
B. obeum A2-162	0.00	0.08	0.40	0.00	0.00	1.00	1.00	0.00	0.20	10.00	0.00	0.00	0.00	0.00	0.00	0.00	0.50	0.50	0.40	0.40
B. obeum ATCC 29174	0.00	0.09	0.40	0.00	0.00	2.00	4.00	0.00	0.08	10.00	0.00	0.00	0.00	0.00	0.00	0.00	4.00	0.10	0.10	5.00
Blautia sp. KLE 1732	0.90	0.09	0.40	0.00	0.00	0.80	0.50	0.00	15.00	0.10	0.00	3.00	8.00	0.00	5.00	0.00	3.00	0.10	0.10	3.00
Blautia sp. Marseille-P3087	0.00	0.85	0.20	0.00	0.00	2.00	4.00	0.00	0.20	0.00	0.00	1.00	0.00	8.00	0.00	5.00	1.00	0.80	0.00	0.50
B. aphidicola str. APS	0.20	0.09	0.00	0.00	40.00	0.00	0.00	0.00	0.10	0.10	20.00	15.00	0.00	0.00	0.00	0.00	0.20	0.00	0.00	0.00
B. crossotus DSM 2876	0.05	0.10	0.08	0.00	0.20	8.00	40.00	0.00	0.70	0.09	0.00	0.50	0.00	0.00	0.00	0.00	0.00	0.30	0.90	0.00
C. jejuni ATCC 700819	0.00	0.10	0.00	0.00	7.00	0.00	0.00	0.00	0.50	0.10	0.00	0.20	0.00	0.00	0.00	0.00	0.10	0.10	0.00	0.10
C. albicans SC5314	0.05	0.10	0.90	0.00	1.00	0.00	0.00	0.00	0.70	0.00	0.00	0.70	0.00	0.00	0.00	0.00	2.00	2.00	2.20	2.00
C. massiliensis JC225	0.08	80.00	0.10	40.00	0.00	0.00	0.00	0.00	0.00	0.05	0.00	0.00	18.00	0.00	2.00	0.00	1.00	8.00	6.00	3.70
C. timonensis SN7	30.00	0.20	0.65	30.00	0.00	0.00	0.00	0.00	0.10	0.00	0.00	0.20	0.00	18.00	0.00	2.00	1.00	1.50	0.10	0.80
C. leptum DSM 753	0.08	0.20	0.30	0.10	0.00	0.20	0.00	15.00	0.10	0.00	0.00	1.00	0.00	0.00	0.00	0.00	0.00	0.00	0.00	0.00
C. eutactus ATCC 27759	0.00	0.90	0.09	0.00	0.00	0.80	1.00	0.00	0.50	0.40	0.00	0.00	0.00	0.00	0.00	0.00	0.00	0.00	0.00	0.00
D. formicigenerans ATCC 27755	0.10	0.40	0.50	0.00	0.00	4.00	8.00	0.00	0.70	0.08	0.00	0.00	0.00	0.00	0.00	0.00	0.00	0.00	0.00	0.00
E. cloacae FDA-CDC-AR_0164	0.00	0.40	0.85	0.50	0.00	0.00	0.00	0.00	0.00	0.10	0.00	0.20	0.00	0.00	0.00	0.00	0.30	0.50	0.20	0.30
E. lavalensis NLAE-zl-G277	0.50	0.00	0.90	1.00	0.00	0.50	0.25	0.00	0.10	0.20	0.00	0.00	0.00	0.00	0.00	0.00	0.80	0.80	0.70	0.70
E. coli K12 substr. MG1655	0.09	0.40	0.80	0.20	0.00	0.00	0.00	0.00	0.10	0.60	0.00	0.40	4.00	0.00	15.00	0.00	0.00	0.00	0.00	0.00
E. coli O157:H7 str. Sakai	0.00	0.40	0.99	0.10	0.00	0.00	0.00	0.00	0.10	0.10	25.00	12.00	0.00	4.00	0.00	15.00	0.00	0.00	0.00	0.00
E. rectale ATCC 33656	0.80	0.60	0.40	0.00	0.00	4.00	8.00	0.00	20.00	0.00	0.00	0.00	0.00	0.00	0.00	0.00	5.00	0.80	0.00	4.50
E. ventriosum ATCC 27560	0.01	0.60	0.08	0.00	0.40	30.00	0.00	0.00	20.00	0.07	0.00	0.00	0.00	0.00	0.00	0.00	8.00	8.00	7.00	8.00
E. peruensis M6.X2	0.60	0.65	0.00	0.00	0.20	0.10	0.00	0.00	0.60	0.20	0.00	6.00	0.00	0.00	0.00	0.00	0.20	7.00	8.00	3.00
F. prausnitzii A2-165	0.01	0.65	0.09	3.00	0.00	0.10	0.00	4.00	10.00	0.00	0.00	0.00	0.00	0.00	0.00	0.00	5.00	5.00	1.00	3.00
F. nucleatum subsp. animalis ATCC 51191	0.02	0.70	80.00	0.00	10.00	0.00	0.00	0.00	0.00	15.00	0.00	4.00	0.00	20.00	0.00	12.00	0.50	0.40	0.20	0.40
F. nucleatum subsp. fusiforme ATCC 51190	10.00	0.70	0.09	0.00	30.00	0.00	0.00	0.00	0.00	15.00	0.00	0.40	20.00	0.00	12.00	0.00	0.50	0.50	0.60	0.50
H. pylori 26695	0.02	0.80	0.00	0.00	0.00	0.00	0.00	0.00	0.50	0.00	0.00	0.00	0.00	0.00	0.00	0.00	0.10	0.00	0.00	0.00
L. acidophilus LA1	0.03	0.80	0.00	0.00	0.50	0.50	0.00	0.00	0.20	0.10	0.00	0.00	0.00	0.00	0.00	0.00	0.40	0.60	0.30	0.60
M. oralis JMR01	0.50	0.80	0.00	0.00	7.00	0.00	0.00	0.00	0.05	0.80	0.00	0.10	4.00	0.00	8.00	0.00	1.00	1.20	0.10	0.70
M. smithii ATCC 35061	0.03	0.85	0.00	0.00	3.00	0.00	0.00	0.00	0.40	0.10	0.00	0.00	0.00	4.00	0.00	8.00	0.00	0.00	0.00	0.00
P. distasonis ATCC 8503	0.10	0.95	0.80	0.00	0.00	0.00	0.00	0.00	0.00	0.10	0.00	0.00	0.00	0.00	0.00	0.00	14.00	8.00	7.00	11.00
R. intestinalis L1-82	0.90	0.95	0.80	0.00	0.00	0.80	1.00	0.00	0.70	0.60	0.00	0.30	0.00	0.00	0.00	0.00	0.00	0.00	0.00	0.00
Rotavirus A	0.00	0.99	0.90	0.00	0.50	0.00	0.00	0.00	0.07	0.70	0.00	0.00	0.00	0.00	0.00	0.00	0.10	0.10	0.00	0.10
R. bromii YE282	0.00	0.99	0.08	0.00	0.00	0.20	0.00	40.00	0.20	0.70	0.00	0.00	0.00	6.00	0.00	6.00	0.00	3.00	4.00	1.00
R. gnavus AGR2154	0.00	0.80	0.60	0.00	0.00	1.00	2.00	0.00	0.60	0.70	0.00	0.00	6.00	0.00	6.00	0.00	0.00	0.10	1.00	0.00
Ruminococcus sp. SR1/5	0.20	0.90	0.50	0.00	0.00	0.20	0.00	25.00	15.00	0.60	0.00	0.20	0.00	0.00	0.00	0.00	2.00	2.00	1.80	2.10
S. variabile DSM 15176	0.15	0.50	0.10	7.00	0.00	0.10	0.00	2.00	10.00	0.50	0.00	0.50	0.00	0.00	0.00	0.00	8.00	0.20	0.00	6.00
T. nexilis DSM 1787	0.25	0.90	0.65	0.00	0.00	6.00	10.00	0.00	0.00	0.50	0.00	0.80	0.00	0.00	0.00	0.00	0.00	0.00	0.00	0.00

Table S4. Summary of genes and bin assignments along the creation of the gold standard. The non-redundant gene catalog corresponds to the representative sequences resulting from the clustering of the set of extracted genes. The gold standard with Single Assignments (GS_SA) comprises only the assignments of the representative genes whereas the gold standard with complete assignments (GS) includes also the species assignments from non-representative genes. For each non-representative gene, its species assignment is added to the GS by associating it to its representative gene only if: (a) the non-representative gene does not belong to the same species of the representative gene of its cluster, (b) another non-representative gene of the same species has not already been added to the GS.

**Gene catalog or gold standard**	**Number of genes/assignments**
Extracted genes	145862
Non-redundant gene catalog	128267
GS_SA (single assignments)	128267
GS (complete assignments)	134338
(a) *Not included in GS*: Assignments from/to the same species	10813
(b) *Partially included in GS*: Assignments from/to a different species	6782

SUPPLEMENTARY FIGURES

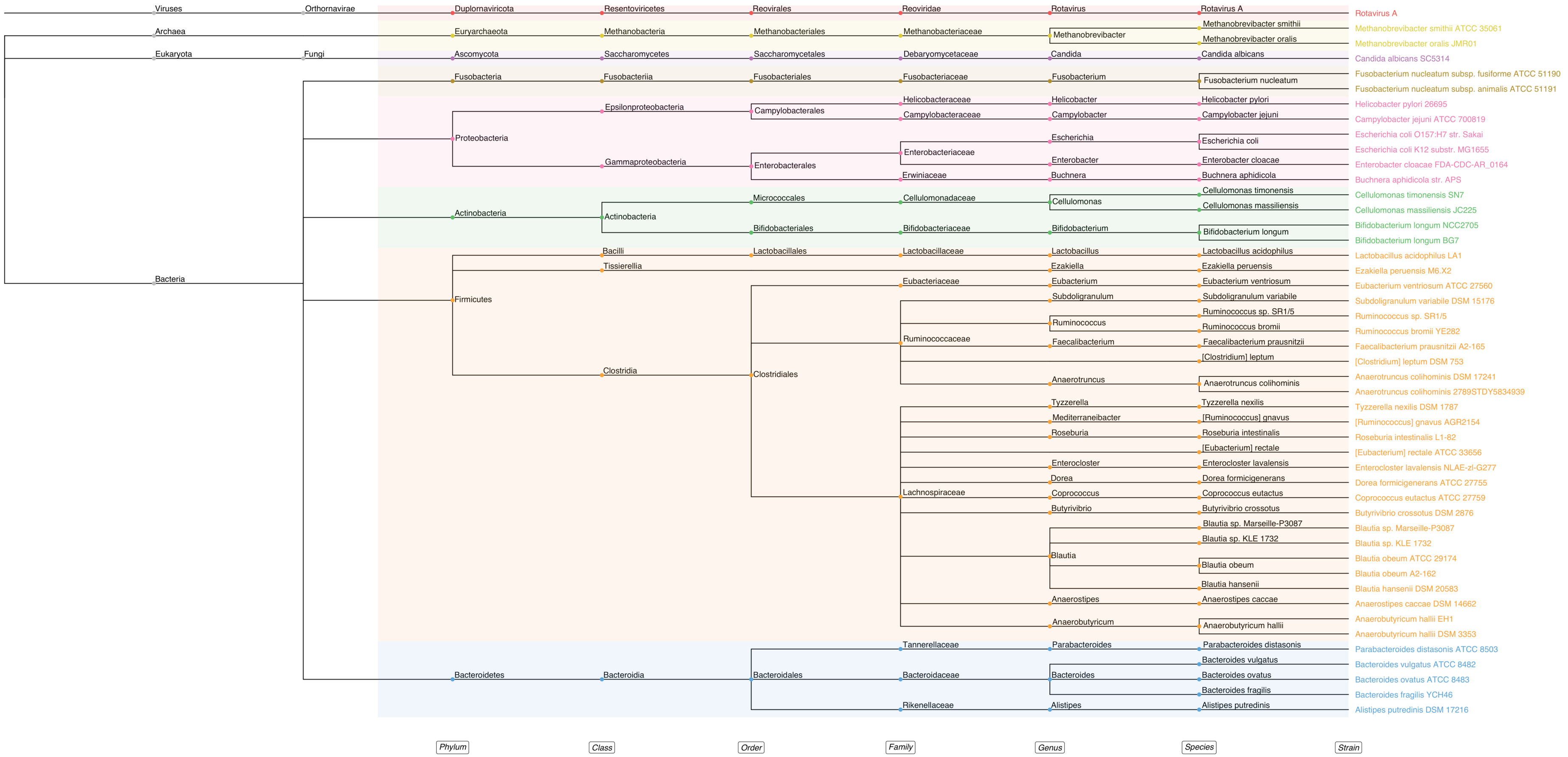

Figure S1. Tree representation of the taxonomy of the strains included in the SGC. This tree representation was computed based on the taxonomy found on the NCBI for each strain. It shows an overview of the taxonomy of the 47 selected strains in relation to each other at the different taxonomic levels. Branches are highlighted with a color specific to each Phylum. For Bacteria, the following colors are represented: Blue – Bacteroidetes; Orange – Firmicutes; Green – Actinobacteria; Pink – Proteobacteria; Brown – Fusobacteria. Archaea, Eukaryotes and Viruses are respectively highlighted with yellow, purple and red colors.

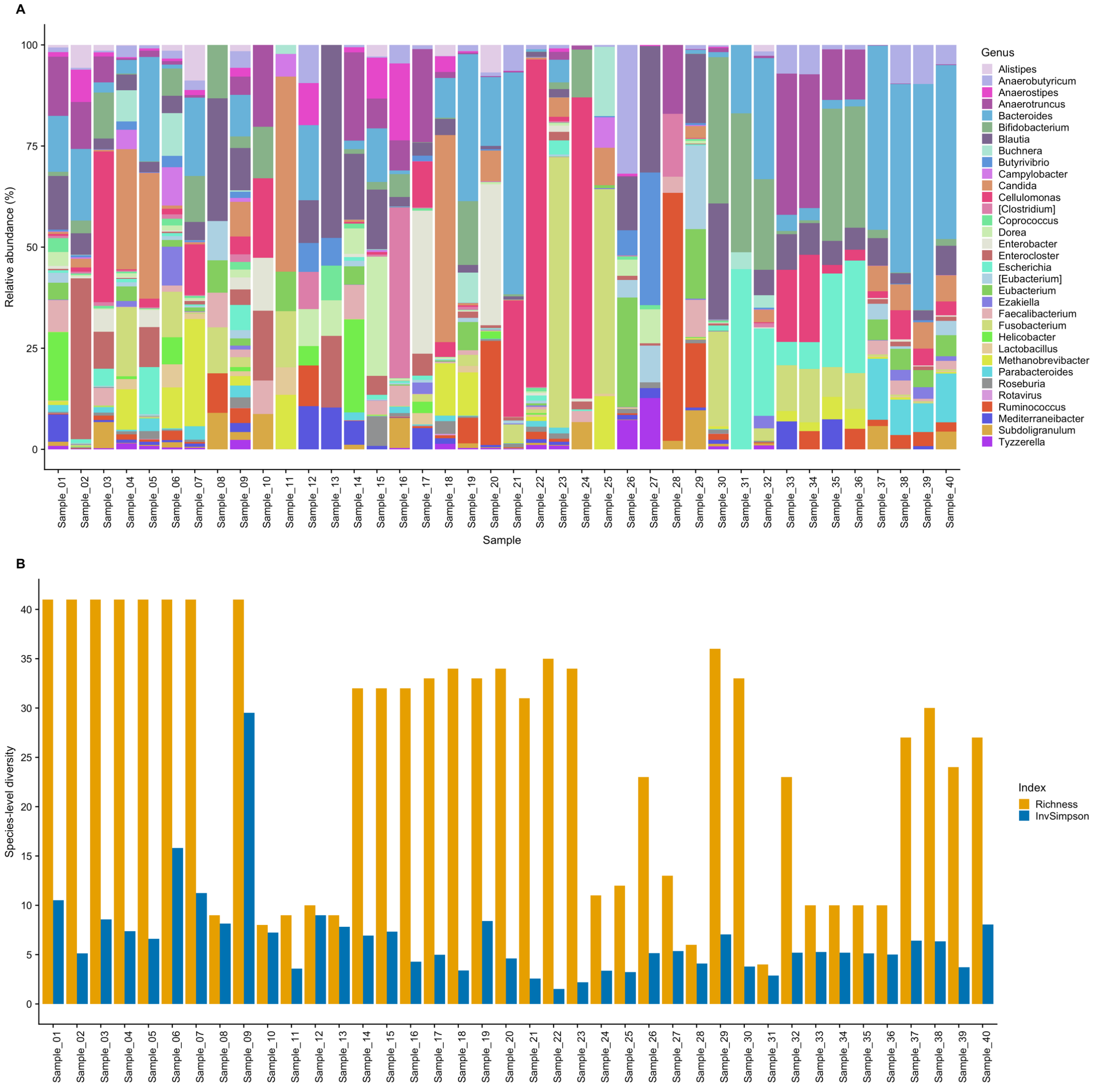

Figure S2. Community structure of the SGC at the genus-level and alpha-diversity at the species-level of the 40 simulated samples.

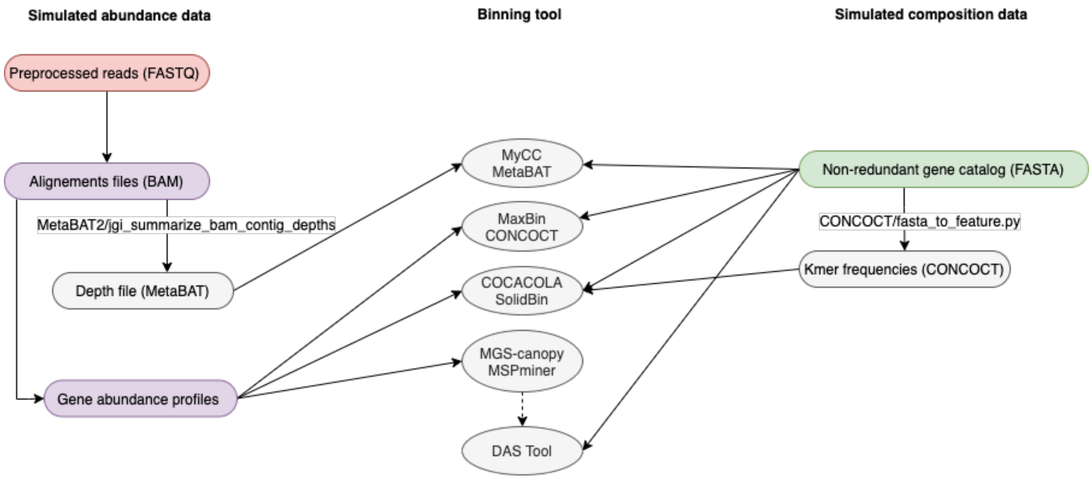

Figure S3. Input requirements for the benchmarked binners. The input of each binner is represented by incoming arrows and is colored according to the step of the construction workflow during which they were created (more details in the legend of Figure 1). This figure represents a simplified view of the required input: data filtering, sorting or file reorganization are not shown. The dashed arrow pointing to DAS TOOL symbolizes that only MGS-CANOPY and MSPMINER have been taken as input.

REFERENCES

1. Nielsen,H.B., Almeida,M., Juncker,A.S., Rasmussen,S., Li,J., Sunagawa,S., Plichta,D.R., Gautier,L., Pedersen,A.G., Le Chatelier,E., *et al.* (2014) Identification and assembly of genomes and genetic elements in complex metagenomic samples without using reference genomes. *Nat. Biotechnol.*, **32**, 822–828.

2. Plaza Oñate,F., Le Chatelier,E., Almeida,M., Cervino,A.C.L., Gauthier,F., Magoulès,F., Ehrlich,S.D. and Pichaud,M. (2019) MSPminer: Abundance-based reconstitution of microbial pan-genomes from shotgun metagenomic data. *Bioinformatics*, **35**, 1544–1552.

3. Wang,Z., Wang,Z., Lu,Y.Y., Sun,F. and Zhu,S. (2019) SolidBin: improving metagenome binning with semi-supervised normalized cut. *Bioinformatics*, **35**, 4229–4238.

4. Lu,Y.Y., Chen,T., Fuhrman,J.A., and Sun,F. (2017) COCACOLA: Binning metagenomic contigs using sequence COmposition, read CoverAge, CO-alignment and paired-end read LinkAge. *Bioinformatics*, **33**, 791–798.

5. Alneberg,J., Bjarnason,B.S., De Bruijn,I., Schirmer,M., Quick,J., Ijaz,U.Z., Lahti,L., Loman,N.J., Andersson,A.F. and Quince,C. (2014) Binning metagenomic contigs by coverage and composition. *Nat. Methods*, **11**, 1144–1146.

6. Kang,D.D., Li,F., Kirton,E., Thomas,A., Egan,R., An,H. and Wang,Z. (2019) MetaBAT 2: an adaptive binning algorithm for robust and efficient genome reconstruction from metagenome assemblies. *PeerJ*, **7**, e7359.

7. Wu,Y.W., Simmons,B.A. and Singer,S.W. (2016) MaxBin 2.0: An automated binning algorithm to recover genomes from multiple metagenomic datasets. *Bioinformatics*, **32**, 605–607.

8. Lin,H.H. and Liao,Y.C. (2016) Accurate binning of metagenomic contigs via automated clustering sequences using information of genomic signatures and marker genes. *Sci. Rep.*, **6**, 24175.

9. Meyer,F., Hofmann,P., Belmann,P., Garrido-Oter,R., Fritz,A., Sczyrba,A. and McHardy,A.C. (2018) AMBER Assessment of Metagenome BinnERs. *Gigascience*, **7**, 1–8.

10. Sczyrba,A., Hofmann,P., Belmann,P., Koslicki,D., Janssen,S., Dröge,J., Gregor,I., Majda,S., Fiedler,J., Dahms,E., *et al.* (2017) Critical Assessment of Metagenome Interpretation - a benchmark of metagenomics software. *Nat. Methods*, **14**, 1063–1071.

11. Pasolli,E., Asnicar,F., Manara,S., Zolfo,M., Karcher,N., Armanini,F., Beghini,F., Manghi,P., Tett,A., Ghensi,P., *et al.* (2019) Extensive Unexplored Human Microbiome Diversity Revealed by Over 150,000 Genomes from Metagenomes Spanning Age, Geography, and Lifestyle. *Cell*, **176**, 649-662.e20.

12. Nayfach,S., Shi,Z.J., Seshadri,R., Pollard,K.S. and Kyrpides,N.C. (2019) New insights from uncultivated genomes of the global human gut microbiome. *Nature*, **568**, 505–510.

13. Almeida,A., Mitchell,A.L., Boland,M., Forster,S.C., Gloor,G.B., Tarkowska,A., Lawley,T.D. and Finn,R.D. (2019) A new genomic blueprint of the human gut microbiota. *Nature*, **568**, 499–504.

14. Sieber,C.M.K., Probst,A.J., Sharrar,A., Thomas,B.C., Hess,M., Tringe,S.G. and Banfield,J.F. (2018) Recovery of genomes from metagenomes via a dereplication, aggregation and scoring strategy. *Nat. Microbiol.*, **3**, 836–843.

15. Strous,M., Kraft,B., Bisdorf,R. and Tegetmeyer,H.E. (2012) The binning of metagenomic contigs for microbial physiology of mixed cultures. *Front. Microbiol.*, **3**, 410.

16. Graham,E.D., Heidelberg,J.F. and Tully,B.J. (2017) Binsanity: Unsupervised clustering of environmental microbial assemblies using coverage and affinity propagation. *PeerJ*, **5**, e3035.

17. Rho,M., Tang,H. and Ye,Y. (2010) FragGeneScan: Predicting genes in short and error-prone reads. *Nucleic Acids Res.*, **38**, e191.

18. Finn,R.D., Clements,J. and Eddy,S.R. (2011) HMMER web server: Interactive sequence similarity searching. *Nucleic Acids Res.*, **39**, W29–W37.

19. Karlin,S., Mrázek,J. and Campbell,A.M. (1998) Codon usages in different gene classes of the Escherichia coli genome. *Mol. Microbiol.*, **29**, 1341–1355.

20. Hooper,S.D. and Berg,O.G. (2000) Gradients in nucleotide and codon usage along Escherichia coli genes. *Nucleic Acids Res.*, **28**, 3517–3523.
